# Bioaccumulation of PFOS Isomers in Transporter Proteins

**DOI:** 10.1021/acs.chemrestox.5c00432

**Published:** 2026-01-08

**Authors:** Deepak James, Jenise Z. Paddayuman, Judith R. Cristobal, Narasimhan Loganathan, G. Ekin Atilla-Gokcumen, Diana S. Aga, Angela K. Wilson

**Affiliations:** † Department of Chemistry and the MSU Center for PFAS Research, 3078 Michigan State University, East Lansing, Michigan 48824, United States; ‡ Department of Chemistry, 12292 University at Buffalo − The State University of New York, Buffalo, New York 14260, United States; § Research and Education in eNergy, Environment and Water (RENEW) Institute, University at Buffalo − The State University of New York Buffalo, New York 14260, United States

## Abstract

The many-decade use of perfluorooctanesulfonic acid (PFOS) in firefighting foams and other products has resulted in their accumulation in water sources and terrestrial environments. Long-term exposure of PFOS has been linked to detrimental effects on human health. PFOS, primarily manufactured through electrochemical fluorination (ECF), yielded both linear and branched isomers. While progress has been made in understanding the health impacts of linear PFOS exposure on human health, there is far less understanding about the toxicological effects and bioaccumulative potential of their branched isomers. In this study, the bioaccumulation potential of linear PFOS and five different branched isomers in the long-chain fatty acid (LCFA) transport protein from *Escherichia coli* (*E. coli*) is investigated using molecular dynamics simulations. The bioaccumulative potential of the PFOS isomers was assessed by computing their binding strength at both the low-affinity site and the high-affinity site in comparison with natural ligands. The binding characteristics of PFOS isomers from *in silico* examinations are in good agreement with *in vitro* cellular studies. Our study demonstrates a preferential bioaccumulation potential for certain branched isomers rather than linear PFOS. The low hydrogen bonding network of disubstituted isomers compared to monosubstituted isomers at the low-affinity site corroborates with their minimal abundance in the *in vitro* studies. The interactions between the PFOS isomers with residues ARG_157 and GLU_319 determine their binding potential. Additionally, the location of −CF_3_ substitutions in branched PFOS isomers plays a crucial role in governing their overall bioaccumulation potential, providing insight about the bioaccumulation potential in living organisms.

## Graphical abstract

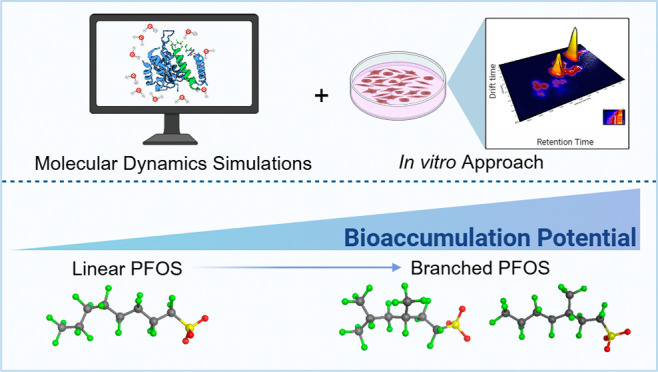

## Introduction

Perfluorooctanesulfonic acid (PFOS) is one of the most important legacy per- and polyfluoroalkyl substances (PFAS), which has been extensively used in aqueous film-forming foam (AFFF) and in various consumer and industrial applications for decades. Due to their thermal and chemical stability,^1,2^ PFOS have been detected in various natural settings including water, soil, sediments, and atmosphere, all of which serve as exposure pathways for humans. Following their environmental persistence,^3–8^ PFOS bioaccumulation^9^ in living organisms has been linked to detrimental health effects, including but not limited to liver toxicity,^10^ immunotoxicity,^11^ endocrine disruption,^12^ and developmental disorders.^13^ As a result, PFOS was added to the list of persistent organic pollutants by the Stockholm Convention in 2009.^14^

Most studies on the bioaccumulative potential of PFOS in humans, animals, and aquatic species have been focused on the investigation of the linear form of PFOS.^15–18^ However, PFOS produced through electrochemical fluorination (ECF) yields a mixture of linear and branched isomers, with average yields of approximately 75 and 25%, respectively.^1,19–21^ Studies on surface water sampling data near contaminated sites in Sweden showed that linear PFOS (L-PFOS) accounted for approximately 80 to 92% of the total PFOS, while branched isomers made up 8 to 20%.^22^ At the same time, sampling data from Norwegian rivers near contaminated sites showed comparable compositions of linear and branched PFOS (Br-PFOS) isomers, with significant variations in composition depending on the time of the year.^23^ Similarly, studies by Ka̋rrman et al.^24^ on soil samples collected from a firefighting training site near the Flesland airport in Norway demonstrated that the total PFOS concentrations were predominantly composed of L-PFOS (63 to 85%), along with a mixture of Br-PFOS isomers. The concentration of L-PFOS in seepage water near contamination sites was significantly lower than in the soil, while branched isomers were found in higher concentrations in seepage water compared to in soil. Similarly, Chen et al.^25^ indicated that the concentration of L-PFOS in both sediment and in various aquatic organisms from Lake Taihu was around 80%, with the remaining 20% consisting of different PFOS isomers. Their findings also showed that the distribution of isomers was substantially higher in surface water than in sediments and that the bioaccumulation of 1m-PFOS (with the −CF_3_ substitution closer to the functional group) was selectively enriched in organisms compared to its concentrations in surface water and sediment. In animal studies on apex predators, an average accumulation of PFOS isomers ranges from 11 to 24%.^21^ In contrast, the bioaccumulation of PFOS isomers in humans ranges from 25 to 51%, suggesting that branched isomers may have a greater bioaccumulative potential in humans.^21^ Also, further insight about the distribution of PFOS isomers in different matrices is provided by Londhe et al.^26^ Therefore, it is evident that certain Br-PFOS isomers preferentially bioaccumulate more than linear ones and that their bioaccumulation potential varies between humans and animals.

Cellular uptake of PFOS in humans is primarily driven by the interactions of PFOS with various proteins, including serum albumin,^27^ fatty acid binding proteins (such as L-FABP and cluster of differentiation [CD36]),^28–30^ and cell membrane transporters.^31,32^ The structural similarity between PFAS and saturated fatty acids enables their binding capabilities with proteins, which facilitates transport to various tissues and disrupts multiple metabolic processes.^33,34^ In cell membranes, PFOS can traverse via two main mechanisms: (i) passive transport through the lipid bilayer^31,35,36^ and (ii) active transport mediated by membrane-bound proteins such as CD36, organic anion transporters (OATs), and organic anion transporting polypeptides (OATPs).^37–39^ Importantly, studies on the cellular uptake of PFOS by OATs/OATPs have shown that the active transport mechanism plays a significant role in PFOS uptake compared to passive transport.^37–39^

Fewer studies have investigated the cellular uptake of L-PFOS via membrane-bound transporters, though some have noted a positive correlation between uptake and carbon chain length.^29,30^ In contrast, only a few studies (*in vitro*, *in vivo*, or epidemiological studies) examined the bioaccumulative potential of branched isomers. For instance, an epidemiological study by Nian et al.^40^ found that the elevated levels of alanine aminotransferase, a marker of liver injury, were positively correlated with Br-PFOS than L-PFOS. Additionally, a study by Tian et al.^41^ indicated that the odds ratio of PFOS isomers associated with overweight status was higher in women (1.33) than in men (0.97), suggesting a sex-dependent impact of Br-PFOS accumulation. Benskin et al.^42^ showed that certain branched sulfonamide precursors were rapidly biotransformed than L-PFOS when incubated with human cytochrome P450 and liver microsomes, thus indicating a potential source for a higher proportion of branched isomers in human serum. Furthermore, a cross-sectional study by Beesoon et al.^43^ on maternal and cord blood serum testing revealed that Br-PFOS crosses the placenta more effectively than L-PFOS. Their study further indicated that isomers with substitutions closer to the functional group (1m-PFOS—0.88: L-PFOS—0.30) exhibited higher transplacental transfer efficiency, as measured by the ratio of PFOS concentration in cord serum at delivery and maternal serum at 15 weeks of gestation.

Despite increasing toxicological studies demonstrating differing bioaccumulation potential for L- and Br-PFOS,^38,44–46^ there is limited mechanistic insight into the factors that dictate their transportation potential in humans. For instance, insight into the intermolecular interactions between different regions of the transporter protein that regulate the movement of L- and Br-PFOS across the cell membrane is inadequate. Therefore, the main objective of the current study is to provide a comprehensive understanding of the bioaccumulative potential of both L- and Br-PFOS isomers. In this study, FadL is used as a model for human fatty acid (FA) transporters to examine the PFAS uptake facilitated by these protein transporters. We first used uptake in human embryonic kidney 293T (HEK293T) cells as a proxy for the bioaccumulative potential of L- and Br-PFOS isomers. We then focused on a fatty acid transport protein to investigate and identify differences in the intermolecular interactions between PFAS and the transporter. The long-chain fatty acid (LCFA) transporter, FadL, is a crucial outer membrane protein in *Escherichia coli* (*E. coli*)^47^ that is essential for LCFA transport.^48^ Studies have shown that FadL exhibits unique, heat-modifiable characteristics^49^ and is substrate-specific for long-chain, not medium-chain, FAs.^50^ Given the lack of structural information about the human LCFA transporter, the crystal structure of FadL from *E. coli* serves as a key model. The structural similarity and hydrophobicity of FAs and PFAS allow FadL to provide insight into the binding interactions between PFAS and FA transporters.^47^

## Methodology

### Ligands

L-PFOS and five Br-PFOS isomers (Figure 1) have been investigated in the current study. Isomers include perfluoro-3-methylheptanesulfonate (P3MHpS), perfluoro-3,5-dimethylhexanesulfonate (P35DMHxS), perfluoro-4,5-dimethylhexanesulfonate (P45DMHxS), perfluoro-5,5-dimethylhexanesulfonate (P55DMHxS), and perfluoro-6-methylheptanesulfonate (P6MHpS). Also, natural ligands (capric acid and palmitic acid) have been investigated to compare their bioaccumulation potential with isoforms of PFOS. Notably, these isomers reported in this study have been detected in seepage water near firefighting training sites.^24^

**Figure 1:**
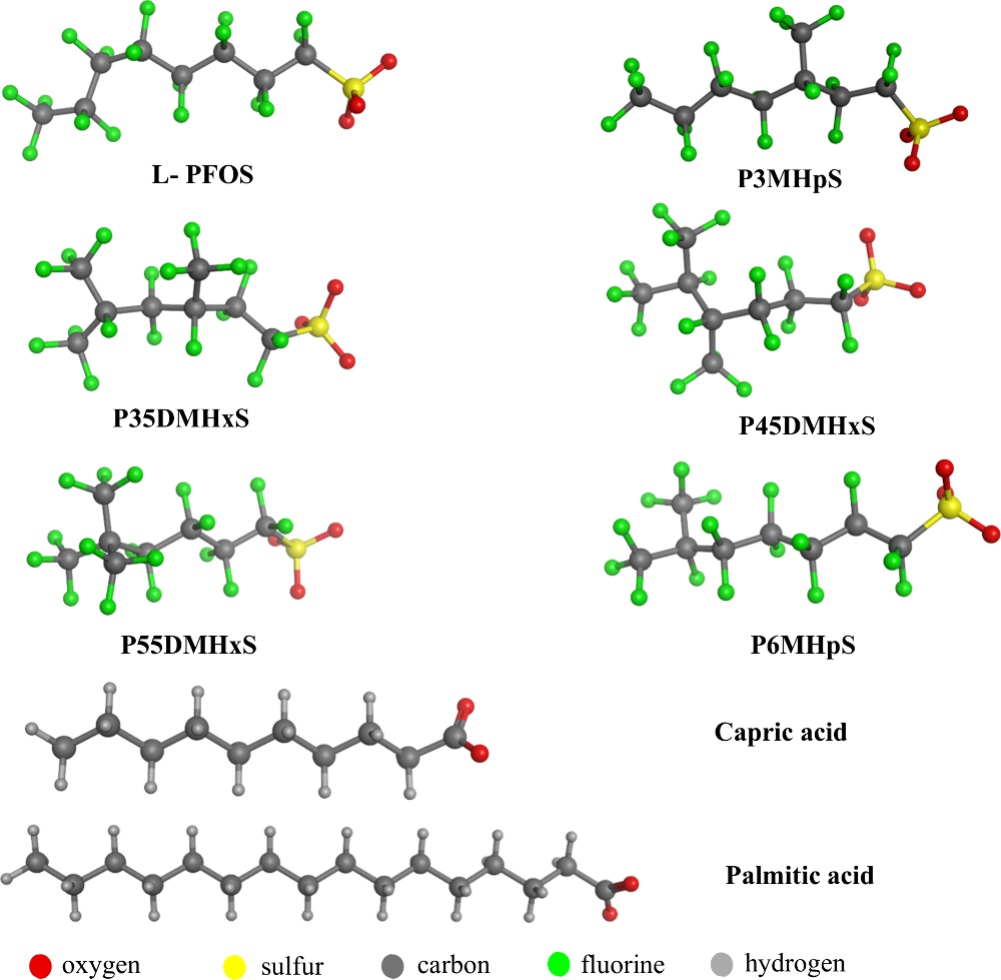
Structural representation of linear and branched isomers of PFOS and natural ligands.

### *In Vitro* Approach

#### Materials

HEK293T cells were purchased from the American Type Culture Collection (Manassas, Virginia). Dulbecco’s modified Eagle medium (DMEM), fetal bovine serum (FBS), penicillin/streptomycin (p/s), and trypsin were purchased from Corning (Corning, New York). Microbicinchoninic (BCA) assay kit was purchased from G Biosciences (St. Louis, Missouri). PFOS was purchased from Millipore Sigma (Burlington, Massachusetts). Ammonium acetate and ammonium hydroxide were purchased from J.T. Baker (Radnor Township, Pennsylvania, and Phillipsburg, New Jersey, respectively). Liquid chromatography–mass spectrometry (LC-MS)-grade methanol (MeOH) and acetonitrile (ACN) were purchased from EMD Millipore (Darmstadt, Germany). Type I water (18.2 MΩ·cm) was generated using a Barnstead Nanopure Diamond (Waltham, Massachusetts) purification system. The BEH C_18_ column, and the data analysis package, DriftScope and UNIFI, were obtained from Waters Corp, Milford, Massachusetts, USA.

##### Cell Culturing, PFOS Exposure, and Cell Collection

The culturing, PFAS exposure, and subsequent collection of HEK293T cells were performed according to a recently published protocol.^29^ Briefly, HEK293T cells were cultured in DMEM supplemented with 4.5 g/L glucose and l-glutamine without sodium pyruvate. The media were supplemented with 10% (v/v) FBS and 1% (v/v) p/s. Cells were grown at 37 °C and 5% CO_2_ until they reached 80–90% confluency for use. Cells were treated with methanol (vehicle control) and 1 μM PFOS in 10 cm dishes (*n* = 3) for 24 h. Cells were collected and centrifuged at 300 relative centrifugal force (rcf) for 5 min at 4 °C. The media were decanted, and the cells were washed three times with cold 1× phosphate-buffered saline (PBS) to remove residual media using the centrifugation protocol previously described. The cell pellet was resuspended in 500 μL of cold 1× PBS. For PFOS isomer analysis, 50 μL aliquots were set aside for protein quantification. Cell pellets were stored at −80 °C until subsequent analysis.

#### PFOS Extraction

PFOS-treated cells were analyzed following a published protocol.^51^ Cell pellets were resuspended in 1 mL of cold methanol and were sonicated using a probe sonicator set at 40% power for 30 s while kept on a cold metal block. Samples were then centrifuged at 16,900 rcf for 12 min at 4 °C. Carefully, 900 μL of the resulting supernatant was transferred to a 1 mL dram vial. The extraction process was repeated with the supernatants; extracts were combined and evaporated to dryness by using a nitrogen evaporator. Final extracts were reconstituted with the mobile phase system at 99:1 (v/v) (A) 5 mM ammonium acetate in type I water pH 3.8 and (B) methanol with 0.1% ammonium hydroxide.

#### Cyclic Ion Mobility Spectrometry (cIMS) Acquisition and Analysis

PFOS isomeric analysis was performed using Waters Acquity Premier UPLC tandem with SELECT SERIES cIMS and quadrupole time-of-flight mass spectrometry (qToF-MS). Liquid chromatography parameters were adapted from an application note^52^ while the cIMS-qToF isomer separation conditions were from an established laboratory protocol.^53^ Analytes with an injection volume of 5 μL were run on a BEH C_18_ column of 1.7 μm (2.1 mm × 100 mm). The gradient profile consisted of 1% (B) ramped to 100% (B) over 16 min and was held for 4 min before returning to the starting mobile phase of a 1% (B), with a total run time of 28 min for each injection. Spectra were obtained in negative mode electrospray ionization with leucine enkephalin as the lock-mass calibrant. PFOS isomers were analyzed using MassLynx, DriftScope, and UNIFI software. Quantification was performed by using an external calibration curve for each isomer that was separated.

### Protein Structure

FadL exists in two crystal forms, monoclinic (PDB ID: 1T16) and hexagonal (PDB ID: 1T1L).^47^ The current *in silico* study is focused on the hexagonal crystal form of FadL protein, which is composed of three major domains (Figure 2): (i) extracellular domain, which features two loops (L3 and L4), one of which is a folded α helix and the other a flexible loop; (ii) transmembrane domain, which connects to the extracellular domain and is composed of 14 antiparallel β-strands forming a long barrel; and (iii) hatch domain, which contains three small helixes (H1, H2, and H3) connected by corresponding loops (L1 and L2). Studies have identified two binding pockets for FadL: the low-affinity site (LAS) and the high-affinity site (HAS). The LAS is a solvent-exposed groove located between L3 and L4 loops, serving as the initial contact point for LCFAs (Figure S1). In contrast, HAS is situated in the extracellular region of the barrel and features three major polar residues, ARG_157, LYS_317, and GLU_319, which likely interacts with the headgroup of fatty acids (Figure S1). To our knowledge, there is currently no available information about residues for LAS. Notably, FadL contains a well-conserved NPA sequence located within the hatch domain. van den Berg et al.^47^ demonstrated that the LCFAs will first bind to LAS and, through passive diffusion, subsequently bind to HAS. This process is followed by a conformational change in both the protein’s hatch domain and ligand, which facilitates the transport of the LCFAs.

**Figure 2:**
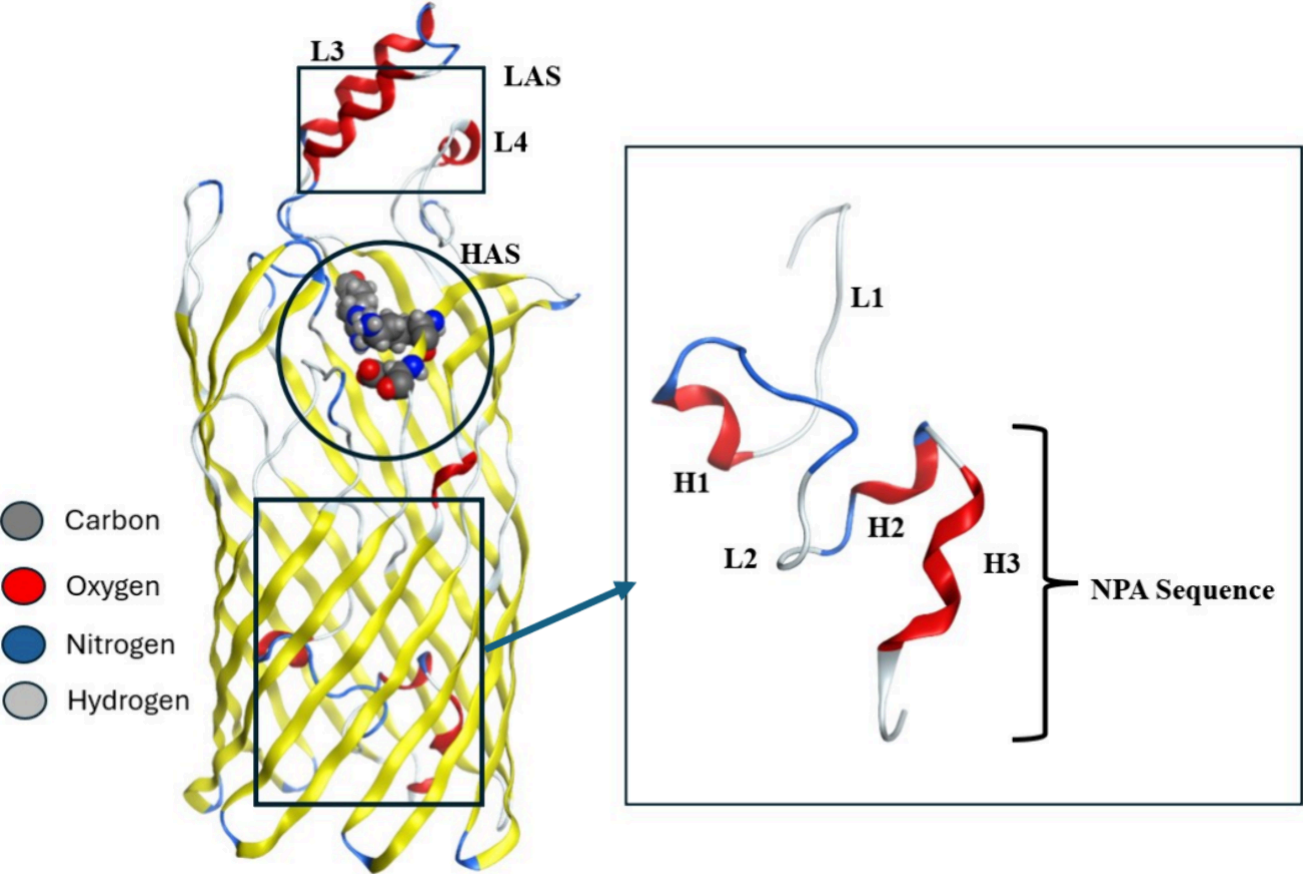
Three-dimensional structure of the hexagonal form of FadL. Important residues at the high-affinity site (HAS) are shown in the ball and stick representation. The hatch domain and the NPA sequence are highlighted on the right side of the FadL structure.

### *In Silico* Approach

#### Molecular Docking

The structure of the hexagonal form of FadL was obtained from the RCSB Protein Data Bank (PDB ID: 1TIL). Molecular Operating Engine (MOE)^54^ was used to protonate the protein^55^ at physiological pH and to identify the potential binding sites within the protein. Based on a prior study, two binding sites were selected for the current study: (i) LAS and (ii) HAS, which is composed of largely polar residues that play a crucial role in protein dynamics.^47^ Initial structures were prepared by placing the ligands in the selected binding sites (LAS/HAS) at physiological conditions, utilizing the Protonate 3D^55^ module (pH 7, 300 K, and 1 atm). These structures were minimized using the AMBER10: Extended Huckel Theory (EHT)^56^ force field in MOE. Induced-fit docking was employed to generate the best possible poses for ligands in the LAS and HAS of the protein. The London Δ*G* ^57^ scoring function was used to rank 100 initial ligand placements, and the top five poses were subsequently refined using Generalized Born Volume Integral/Weighted Surface Area scoring functions (GBVI/WSA).^58^ The final poses for each examined PFOS isomers were selected based on two criteria: (i) the specific orientation of the functional group toward the aforementioned polar residues and (ii) the docking score.

#### Pose Selection

The Triangle Matcher algorithm was employed for pose generation in the LAS pocket. Based on previous studies on natural ligands (dodecanoic acid) and a proposed hypothesis about LCFA transport,^59,60^ the selected poses have a configuration where the sulfonate group of PFOS is pointed outward between the L3 and L4 loops. For the HAS site, a pharmacophore model was utilized to orient the functional groups of ligands toward ARG_157 and LYS_137, in accordance with van den Berg et al.^47^ (Figure S1). The poses with the highest docking scores (lowest negative value) were then selected for molecular dynamics (MD) simulation.

#### Simulation Methodology

MD simulations were carried out as follows: Initial models for all the selected protein–ligand poses at both binding sites (LAS and HAS) were prepared using the CHARMM-GUI^61–63^ solution builder. The topology and parameter files for the protein–ligand complex were generated using CHARMM-GUI, which will be used for running simulations with the AMBER simulation package. The AM1-BCC^64^ charging protocol was used to calculate the partial charges of ligand atoms. The protein–ligand complex was solvated in an orthogonal box and neutralized in 100 mM NaCl.^65^ ff19SB,^66^ GAFF2,^67^ and OPC^68^ interaction potentials were used to describe the intermolecular interactions for the protein, ligands, and water, respectively. Joung and Cheatham parameters^65^ are used for ions. MD simulations were performed with AMBER 2020 ^69^ using the *pmemd* module with CUDA. The minimization was carried out using a series of harmonic potentials (100.0, 50.0, 10.0, and 0.0 kcal mol^–1^ Å^–2^) applied to constrain the protein and ligand, while water molecules and ions were unconstrained. The minimized system was heated in a stepwise manner from 0 to 293.15 K for 3 ns in total, during which the restraints were gradually removed. Subsequently, the systems were equilibrated for 1 ns in the NPT ensemble. Langevin thermostat^70^ and isotropic Monte Carlo barostats^71^ were used to control both temperature and pressure during the simulation.

The SHAKE^72,73^ algorithm was used to constrain the hydrogen bonds of water molecules. A time step of 1 fs was used to integrate the equation of motion, with a cutoff distance of 9 Å for short-range electrostatics, while particle-mesh Ewald (PME)^74^ was employed for long-range interactions. For all PFOS isomers, production simulation runs were performed for 100 ns and duplicated for statistical accuracy. Four different analyses were performed to examine the stability of the ligand, their structural flexibility, and relevant interactions within the protein–ligand complex. Root mean square deviation (RMSD) and root mean square fluctuation (RMSF), hydrogen bonding, and residue decomposition were determined using the CPPTRAJ^75^ module implemented in AmberTools21.

#### Calculation of Binding Energy

The binding free energies for all ligands under investigation were calculated using molecular mechanics with a Poisson–Boltzmann surface area (MM-PBSA) in AMBER 2020.^76^ For the MM-PBSA calculations, the production run of each PFOS isomer was divided into five time-equivalent blocks of 20 ns each. Binding energetics were computed by using frames extracted at 10 ps intervals across the entire simulation run (100 ns). The binding energies were computed using the block averages, and the associated uncertainties were calculated using the single trajectory approach implemented in AMBER.^69^ In addition, per-residue decomposition analysis was performed to determine the energetic contributions of key residues that influence ligand binding at the binding site.

## Results

Given the environmental presence of different PFOS isomers, we sought to compare their bioaccumulation potential. To achieve this, we first conducted uptake experiments in cultured cells using a commercially available PFOS mixture containing both isomer types followed by MD simulations to explore potential mechanistic underpinnings. To investigate the uptake and distribution of PFOS isomers, including L-PFOS and Br-PFOS, we conducted an *in vitro* cellular study. This approach allows us to directly assess the isomer-specific accumulation of the PFOS mixture, thereby minimizing confounding factors typically found in more complex biological systems. We first analyzed the composition of the PFOS mixture and confirmed the isomer distribution in the commercial standard (Figure 3A), which is composed of 85.31% L-PFOS and 14.68% Br-PFOS isomers (Figure 3A). The branched fraction consisted of monosubstituted isomers (P1MHpS, P3MHpS, P4MHpS, P5MHpS, and P6MHpS) as well as disubstituted isomers (P35DMHxS, P45MDHxS, and P55DMHxS). We then exposed HEK293T cells to 1 μM (500 ppb) of the PFOS standard. After 24 h of exposure, we collected the cells, extracted PFOS for analysis using UHPLC–cIMS–qToF–MS, and quantified the isomers via external calibration curves.^53^

**Figure 3:**
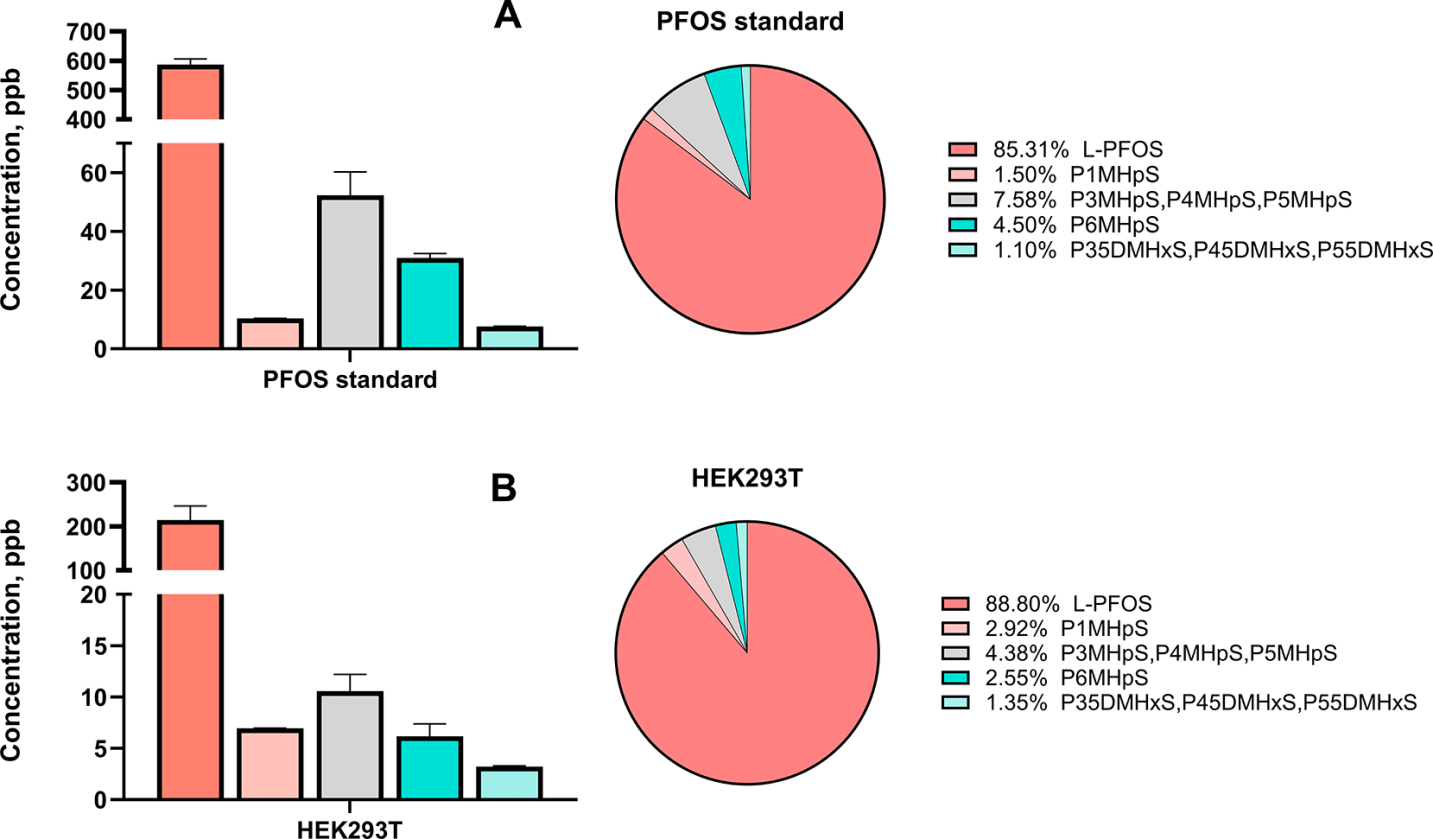
Concentration and percent distribution of PFOS isomers, linear or branched, in reference standard prior to cell exposure (A) and in HEK293T cells (B) following a 1 μM PFOS exposure for 24 h. Data are represented as the means of three independent replicates.

As shown in Figure 3B, HEK293T cells exhibited a preferential uptake of the L-PFOS isomer, which reached a concentration of 194.45 ppb, corresponding to 88.80% of the total intracellular PFOS. This enrichment indicates that the linear form is selectively accumulated relative to the branched counterparts. Among the Br-PFOS group, the predominant isomers were the coeluting P3MHpS, P4MHpS, and P5MHpS, which together accounted for the largest fraction of the branched isomers. In contrast, P1MHpS and P6MHpS were taken up at lower but comparable levels, suggesting that positional branching has a modest impact on the cellular uptake efficiency. The disubstituted PFOS isomers (P35DMHxS, P45MDHxS, and P55DMHxS) displayed the lowest intracellular abundance, confirming their reduced accumulation relative to both linear and monosubstituted PFOS isomers. Overall, these results demonstrate stereoselectivity in PFOS uptake, with L-PFOS being favored, followed by a hierarchy of branched isomers where monosubstituted forms are taken up more efficiently than disubstituted species.

### Binding Free Energy Calculations

To evaluate this isomeric-specific uptake in cells, binding affinities of L- and Br-PFOS were computed at both the LAS and HAS of FadL using MM-PBSA. At LAS, P3MHpS exhibited a higher binding affinity (∼−30 kcal mol^–1^) compared to all PFOS isomers and natural ligands, as it maintains a more attractive interaction with neighboring residues (Table S1). When comparing PFOS isomers, all disubstituted forms exhibited similar binding energies (∼−26 kcal mol^–1^). In contrast, L-PFOS showed lower binding energies than other PFOS isomers, which clearly indicates that substitution causes a significant change in the binding efficiency to FadL (Figure 4). Among natural ligands, palmitic acid shows higher binding energy than capric acid at LAS. The lower binding energy of capric acid could be attributed to their lower vdW energetic contribution toward the overall binding energy (Figure S12). Nevertheless, it is evident from Figure 4 that P3MHpS exhibits a stronger binding potential than natural ligands.

**Figure 4:**
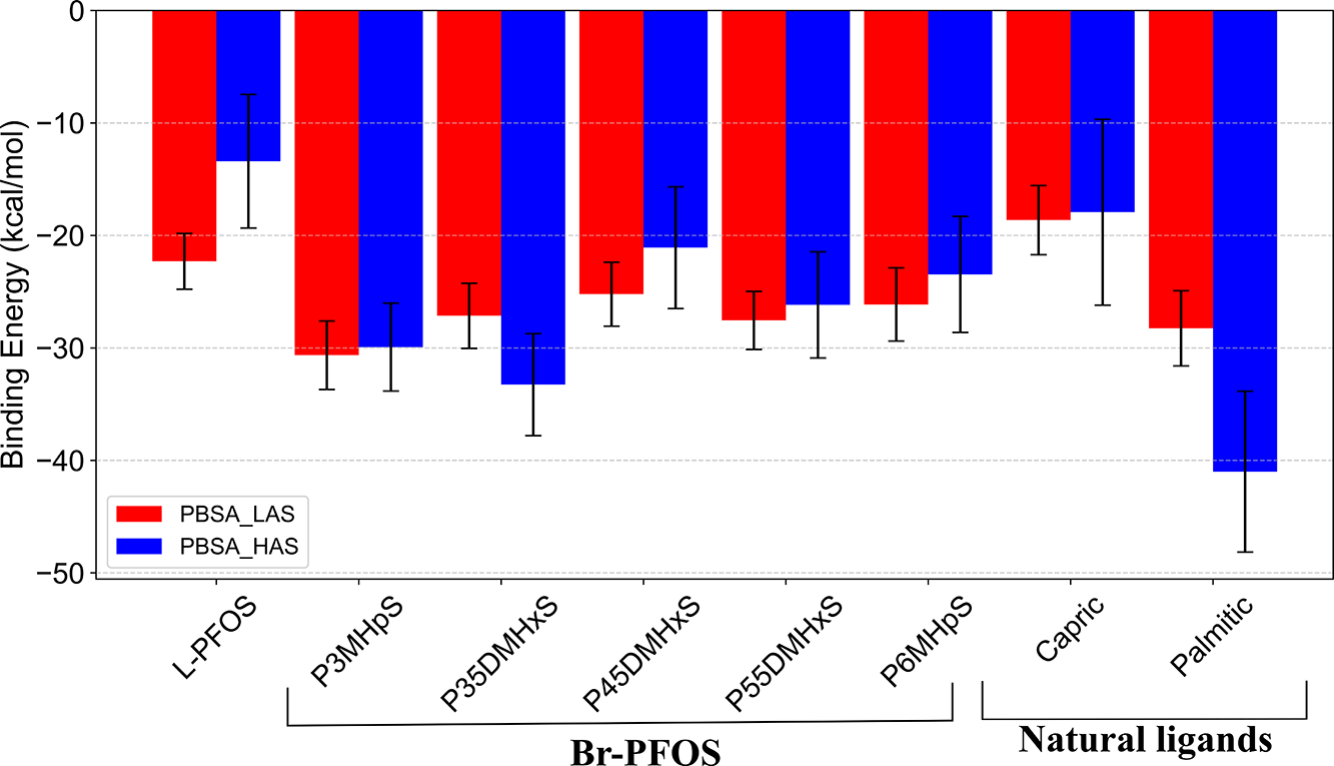
Binding free energy of ligands investigated at the low-affinity site (LAS) and the high-affinity site (HAS).

At HAS, the palmitic acid showed a higher binding energy as compared to the L- and Br-isomers of PFOS, and capric acid. The strong affinity of palmitic acid could be attributed to its ability to exhibit attractive interactions with many neighboring residues of the HAS. (Table S2). Similar to LAS, L-PFOS showed a lower binding energy when compared to Br-PFOS isomers, which is primarily due to the presence of a strong cumulative repulsive interaction between L-PFOS with specific neighboring residues listed in Table S2. This effect was particularly pronounced with residues ASP_365, ASP_363, and THR_268, which were uniquely involved in strong repulsive interactions with L-PFOS. By comparing PFOS isomers, P35DMHxS has a higher binding energy (∼−33 kcal mol^–1^), followed by P3MHpS (∼−30 kcal mol^–1^). In this case, capric acid shows a lower binding energy than all ligands except L-PFOS.

### Per-Residue Decomposition

The per-residue decomposition data (Tables S1 and S2) indicated that the residues at the LAS minimally contributed to ligand binding with energies around −1 to −2 kcal mol^–1^ and are likely due to the presence of fewer polar residues at LAS. Notably, natural ligands exhibited even weaker interactions with the residues compared to the PFOS isomers. On the other hand, at HAS, most of the investigated ligands exhibit attractive interactions with ARG_157, LYS_317, and ILE_155 because these residues are positively charged at pH 7, and the investigated ligands are negatively charged. Compared to L-PFOS, most Br-PFOS isomers exhibited a stronger attractive interaction with ARG_157. In addition, among PFOS isomers, P35DMHxS exhibited a stronger attractive interaction with LYS_317 (∼−4.4 kcal mol^–1^). In addition, natural ligands demonstrated a much stronger attractive interaction with similar residues. On the other hand, both ASP_122 and GLU_319 exhibited repulsive interactions with most ligands, with L-PFOS showing a notably strong interaction of ∼6 kcal mol^–1^ with GLU_319.

### Hydrogen Bonding

The hydrogen bonding interactions were significantly different when comparing LAS and HAS.

Since LAS is composed of predominantly hydrophobic residues and fewer polar residues, no significant hydrogen bonding patterns were seen for PFOS isomers. Two out of the six PFOS isomers exhibited moderate hydrogen bonding with specific residues of LAS. For instance, P3MHpS isomer-maintained hydrogen bonding with two residues: (i) the amide group of ASN_244 for an average of 55% during the simulation run and (ii) ARG_245 for ∼40% during the production run. From Table S1, P3MHpS is the only isomer that maintains an attractive interaction with ARG_245 and shows a stronger attractive contribution from ASN_244 (−2 kcal/mol). Similarly, the PFOS-disubstituted P35DMHxS exhibited hydrogen bonding interactions with ASN_244 at an average of ∼40%. In contrast, natural ligands did not show any significant hydrogen bonding at the LAS (Figure 5).

**Figure 5:**
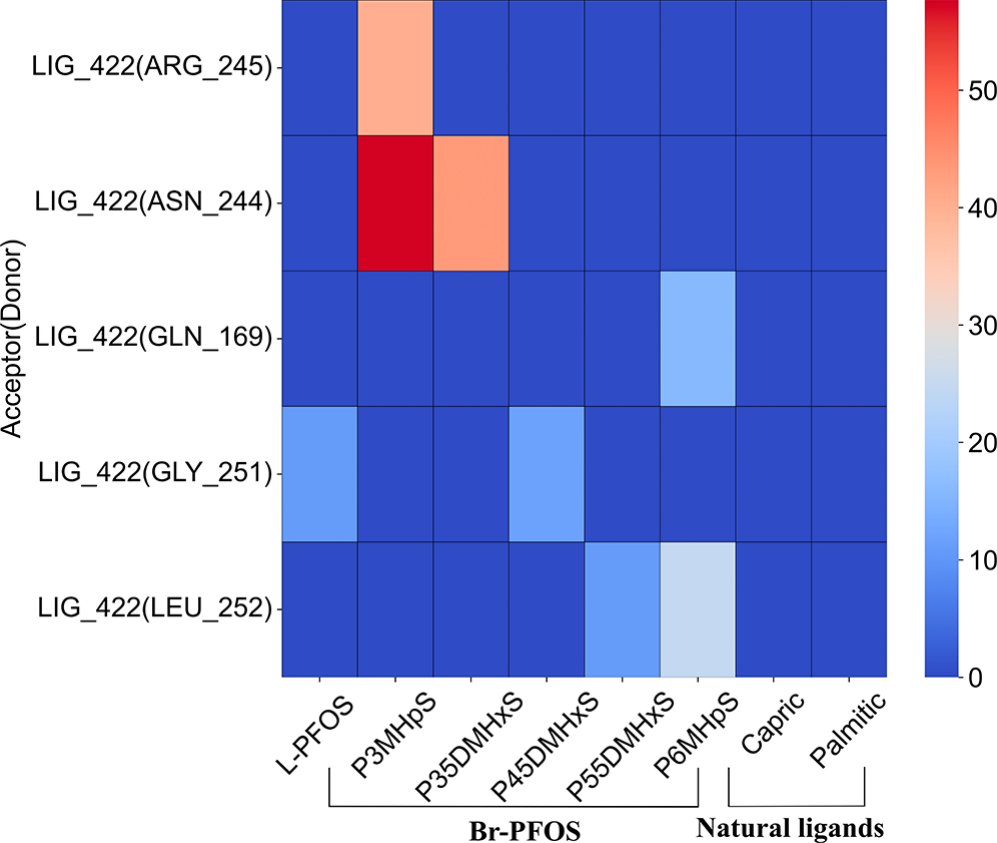
Hydrogen bonding of the ligands at the low-affinity site (LAS).

At HAS, all PFOS isomers exhibited substantial hydrogen bonding interactions due to the higher presence of acidic and basic residues. Among the investigated PFOS isomers, all except L-PFOS demonstrated hydrogen bonding interactions with ARG_157, ranging from 58 to 100%. Such strong interactions of Br-PFOS isomers with ARG_157 may be attributed to the hydrogen bonding between the positively charged guanidinium group and anionic PFAS at physiological pH. Additionally, both capric acid and palmitic acid exhibited strong hydrogen bonding with ARG_157, which is supported by their attractive interaction with ARG_157. The attractive interactions indicated in the per-residue decomposition profile support the ARG interaction, where capric and palmitic acids have interaction energies of approximately −10 and −7 kcal mol^–1^, respectively (Table S2). Furthermore, the amino group of LYS_317 also serves as a hydrogen bond donor for some ligands. Especially, the P35DMHxS isomer showed the highest hydrogen bonding fraction (∼67%) while both L-PFOS and P3MHpS showed the lowest (∼19%). Moreover, the hydroxyl groups of Serine 358 and 360 (SER_358 and SER_360) maintained hydrogen bonding interactions (∼15 to 40%) with most of the ligands (Figure 6 and Figure S13).

**Figure 6:**
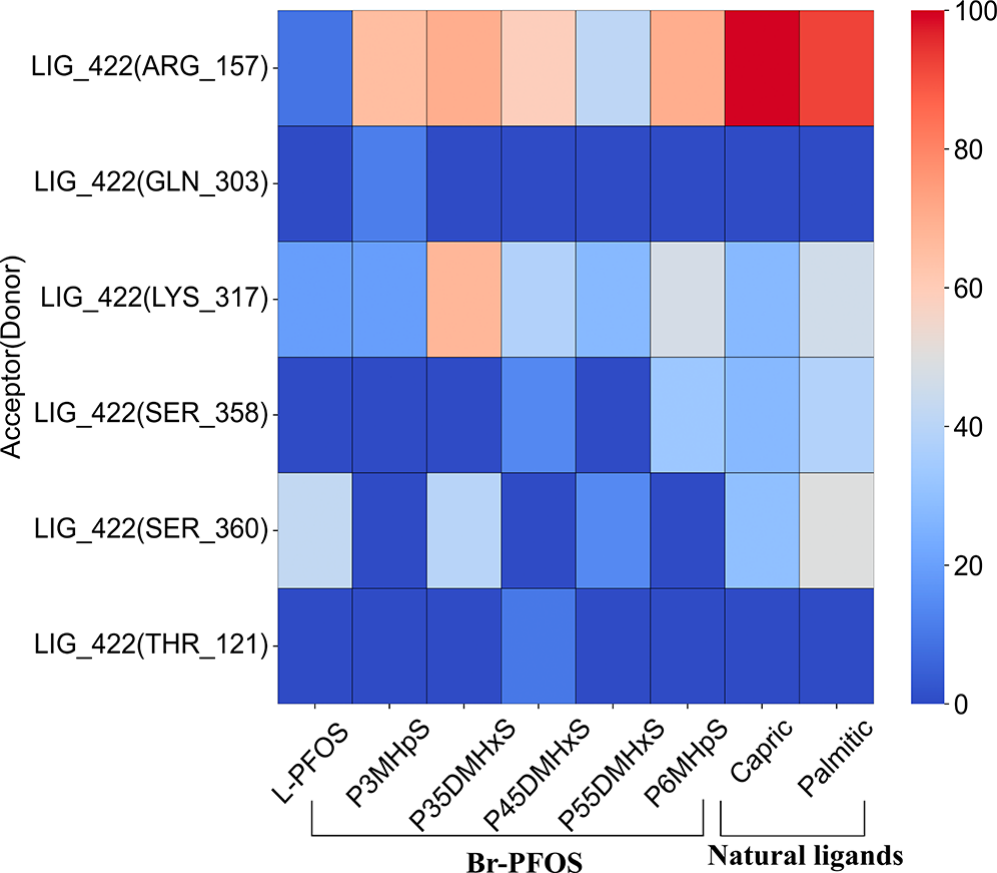
Hydrogen bonding of investigated ligands at a high-affinity site (HAS).

Figure S13 shows the hydrogen bonding details corresponding to the NPA sequence in the FadL hatch domain. The hydrogen bond profiles indicate that the hatch NPA sequence (residues 33–35) is conserved in FadL. The amide side chain of Asparagine 33 (ASN_33) forms a hydrogen bond with the carbonyl backbone of Glycine 21 (GLY_21), which plays a crucial role in stabilizing the hatch domain. The absence of such hydrogen bonds leads to a conformational change in the hatch region and subsequently leads to ligand transport. At both LAS and HAS, noticeable differences in the hydrogen bonding patterns between these residues are observed. Our studies also show a strong hydrogen bonding interaction between ASN_33 and GLY_21 at HAS. This change in the bonding pattern is influenced by the key residues at HAS, which affect the allosteric communication pathways within the protein.

### Synergistic Outcomes from *In Vitro* and *In Silico* Studies

The binding characteristics of L-PFOS and Br-PFOS isomers from MD simulations align well with the *in vitro* experimental data reported in this study, despite slight temperature variations. To validate that temperature has a minimal effect, MD simulations were conducted at 310 K to assess the binding free energies and hydrogen bonding patterns of the L-PFOS and Br-PFOS isomers. The results indicate that the binding energy trends of PFOS isomers are similar between both temperatures (293 and 310 K), with only minor fluctuations in the hydrogen bonding interactions. (see the Supporting Information for details). Our studies clearly indicate that the monosubstituted PFOS isomer (P3MHpS) exhibits higher bioaccumulation potential when compared to isomers that have substitutions far away from the terminal functionality. In addition, based on the binding energetics and intracellular abundance profiles, our studies indicate a higher bioaccumulation potential for monosubstituted PFOS isomers than disubstituted ones.

## Conclusions

The bioaccumulative potential of L-PFOS and five environmentally relevant isomers was investigated using a combination of *in vitro* assays and molecular modeling approaches. The examined isomers include either mono- or disubstitutions of the −CF_3_ group positioned proximally or distally relative to the sulfonate group.

An *in vitro* system was used to compare the bioaccumulation potential of L-PFOS versus Br-PFOS, and it was found that L-PFOS exhibited a greater propensity to bioaccumulate, as determined by cell uptake studies. To gain mechanistic insights into these differences, MD simulations were performed for both L- and Br-PFOS, along with endogenous (natural) FadL ligands and capric and palmitic acids. Binding affinities for all PFOS isomers were calculated for the LAS and HAS sites of the FadL protein.

The simulation results showed that the P3MHpS isomer exhibited higher binding energy than L-PFOS, whereas the disubstituted isomers displayed comparable but slightly elevated binding energy relative to L-PFOS. These computational findings are consistent with the *in vitro* data, reinforcing the notion that structural branching influences the bioaccumulation propensities of PFOS isomers. The per-residue decomposition data indicated that ARG_157 plays an important role in attractive contribution toward most of the ligands, with the strongest for palmitic acid. On the other hand, GLU_319 acts as a repulsive energetic contributor for all ligands, with a value for L-PFOS. The hydrogen bonding profiles at LAS are lower for the disubstituted PFOS by comparing the monosubstituted PFOS. This observation is consistent with *in vitro* studies, where the intracellular abundance of disubstituted PFOS was lower compared to the monosubstituted PFOS. As shown in this work, PFOS isomers can vary in their accumulative behavior. When there is a −CF_3_ branch close to the headgroup of PFOS, a higher bioaccumulative potential can be exhibited than for the linear PFOS. Thus, there is a need to understand the bioaccumulation potential of different PFOS isomers in humans and animals.

## Supplementary Material



## Notes

Schematic of LAS and HAS, RMSDs, RMSFs, hydrogen bonding profiles, and per-residue decomposition details of PFOS isomers in FadL; discussion on the results as a function of two different temperatures (PDF)
