# Supplementary material for: Bioaccumulation of PFOS Isomers in Transporter Proteins
Source: Chem Res Toxicol. 2026 Jan 8;39(1):168–77. doi: 10.1021/acs.chemrestox.5c00432 (PMC12820963; doi:10.1021/acs.chemrestox.5c00432)
Supplement: Supplementary file 1 [file tx5c00432_si_001.pdf]

Supplementary Information for

Bioaccumulation of PFOS Isomers in Transporter Proteins

Deepak James,[a] *Jenise Z. Paddayuman,*[b] *Judith R. Cristobal,*[b,c] *Narasimhan Loganathan,*[a] *G. Ekin Atilla-Gokcumen,*[b] *Diana S. Aga,*[b,c] *and Angela K. Wilson*[a*]

[a)] Department of Chemistry and the MSU Center for PFAS Research, Michigan State University, East Lansing, Michigan 48824, United States

[b)] Department of Chemistry, University at Buffalo – The State University of New York, Buffalo, New York 14260, United States

[c)] RENEW Institute, University at Buffalo – The State University of New York (SUNY), Buffalo, New York 14260, United States

Corresponding author: akwilson@msu.edu

Contains Figures – 17 and Tables – 4

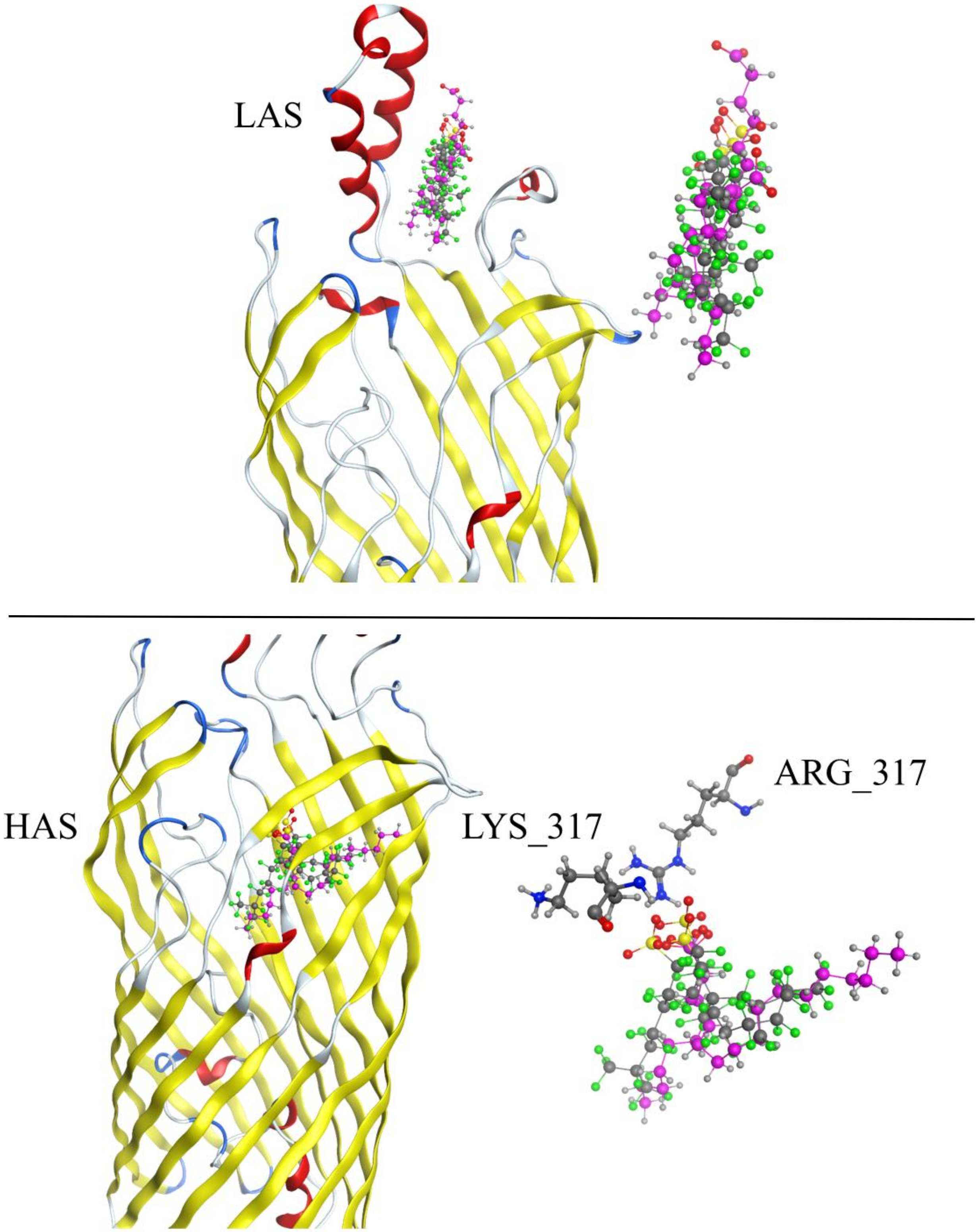

<u>Figure S1:</u> A three-dimensional representation of low-affinity site (LAS) and high-affinity site (HAS), two binding sites of FadL, along with the superimposed ligands and directionality of the functional group.

RMSD

Irrespective of the ligands at LAS, FadL remained stable throughout the simulation run. For most of the PFOS isomers examined, the root mean square deviation (RMSD) profiles indicated that the protein backbone was stabilized within 20–40 ns. The RMSD values of ~ 0.8-2.5 Å demonstrate minimal deviation from the reference structure (i.e., initial frame of production run) even in the presence of PFOS (**Figures S2-S9**). On the other hand, at HAS, the protein stabilized within 20-30 ns of simulation time. The RMSD for all PFOS isomers at HAS shows smaller fluctuations (1 to 2 Å) than their corresponding RMSD values at LAS, which could be attributed to the strong interaction between the ligands and the polar residues at HAS.

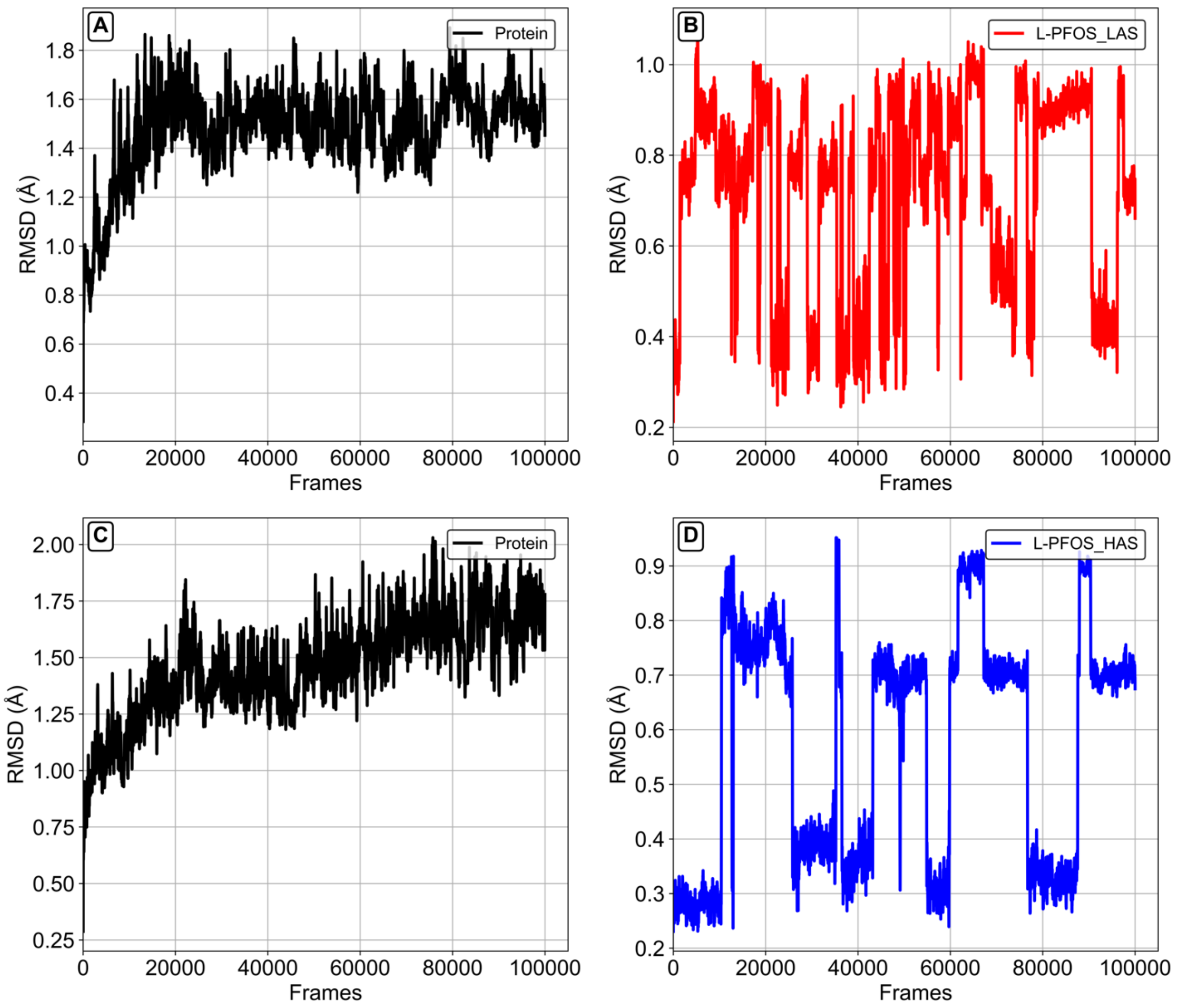

Figure S2: RMSD of Protein (A, C) and L-PFOS (B, D) RMSD at LAS and HAS.

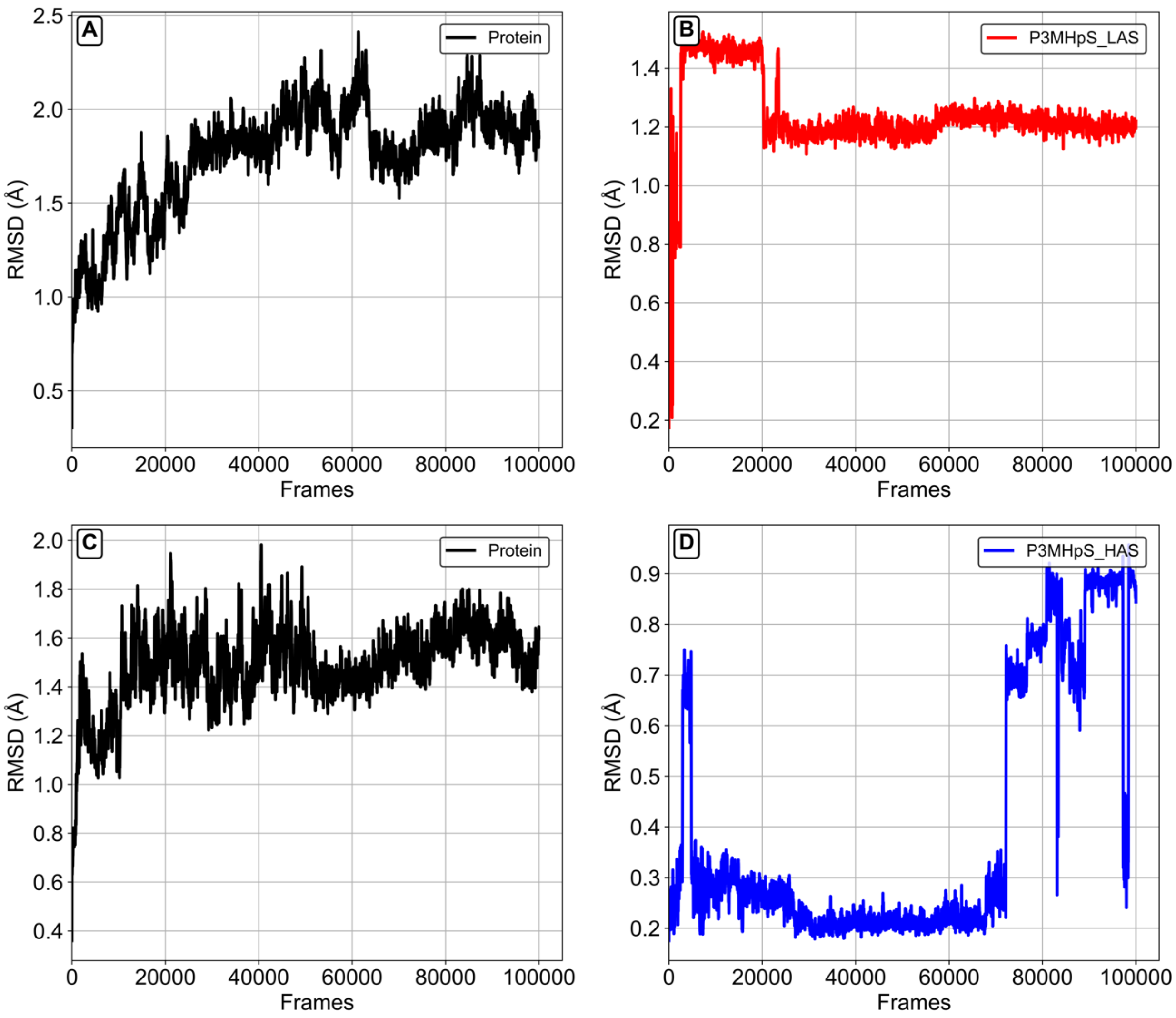

<u>Figure S3</u>: RMSD of Protein (A, C) and P3MHpS (B, D) RMSD at LAS and HAS.

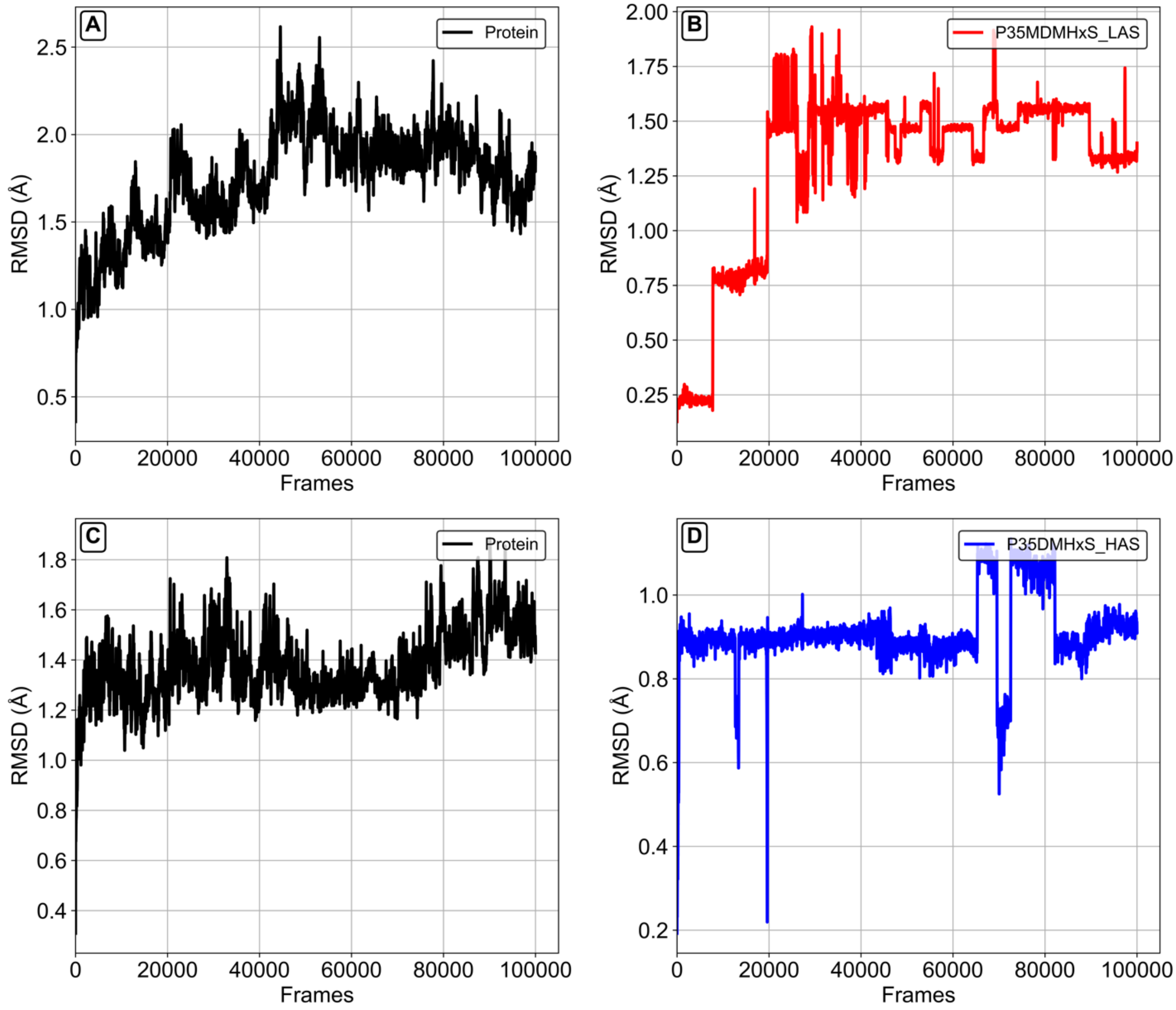

Figure S4: RMSD of Protein (A, C) and P35DMHxS (B, D) RMSD at LAS and HAS.

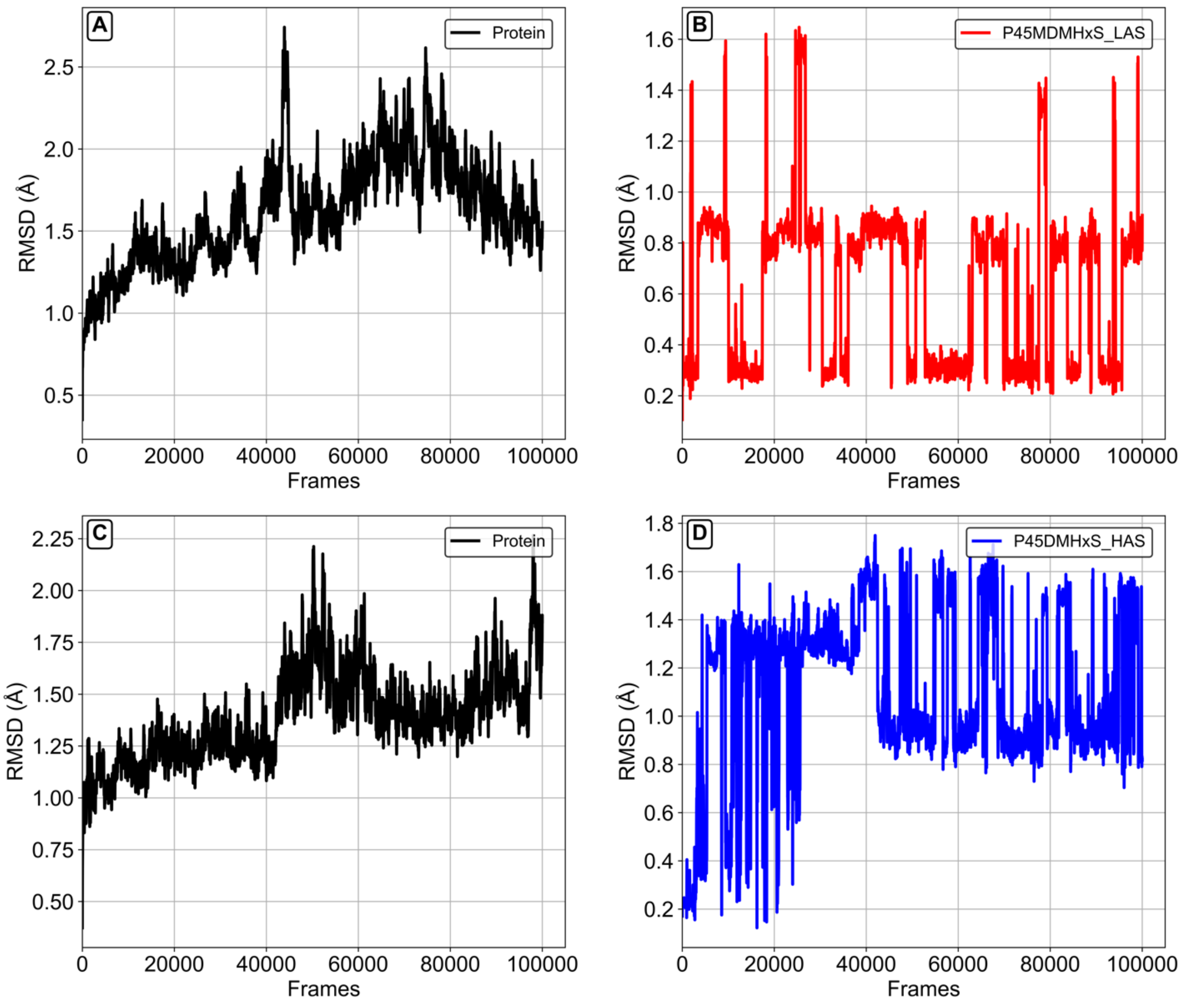

<u>Figure S5</u>: RMSD of Protein (A, C) and P45DMHxS (B, D) RMSD at LAS and HAS.

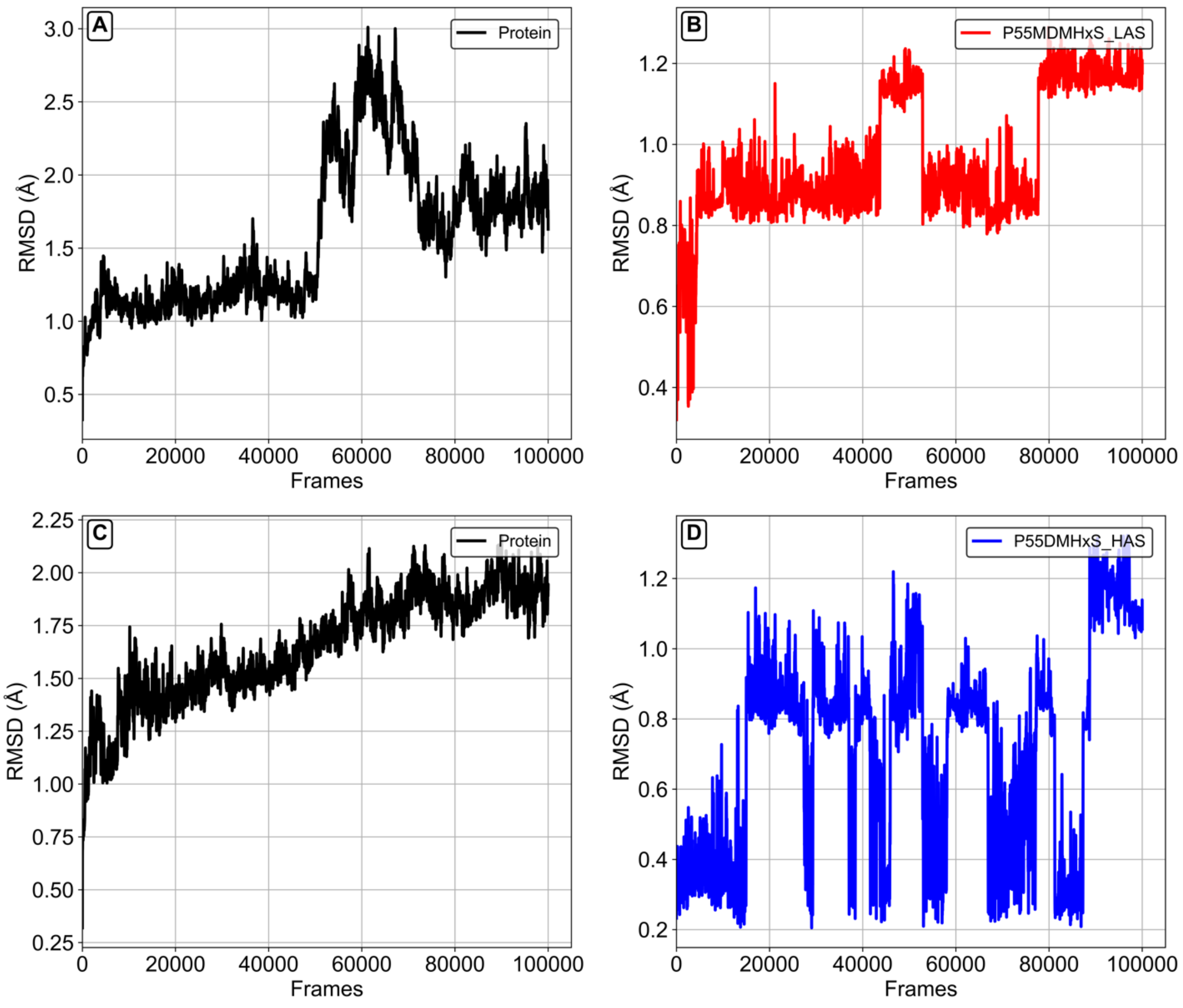

Figure S6: RMSD of Protein (A, C) and P55DMHxS (B, D) RMSD at LAS and HAS.

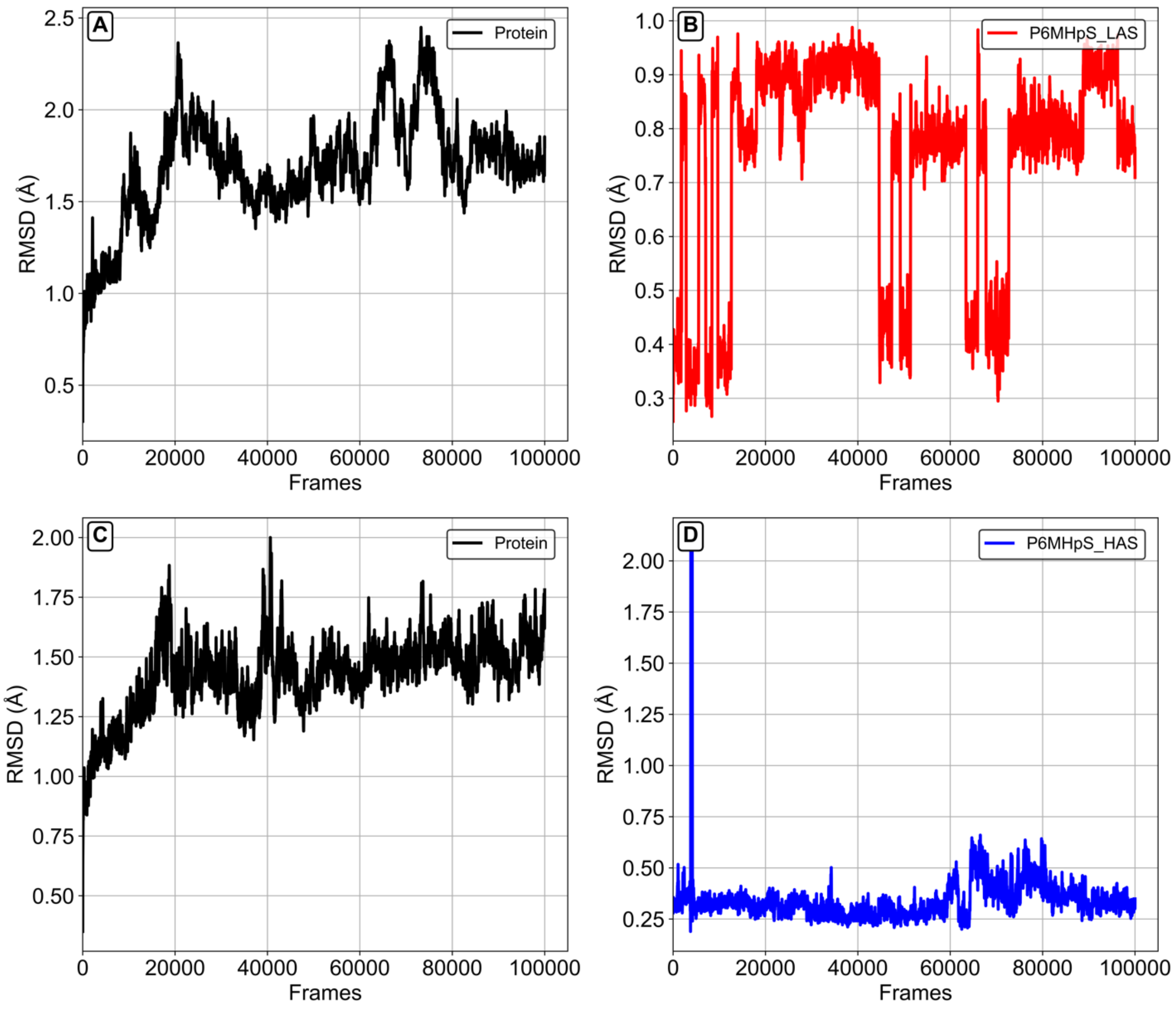

Figure S7: RMSD of Protein (A, C) and P6MHpS (B, D) RMSD at LAS and HAS.

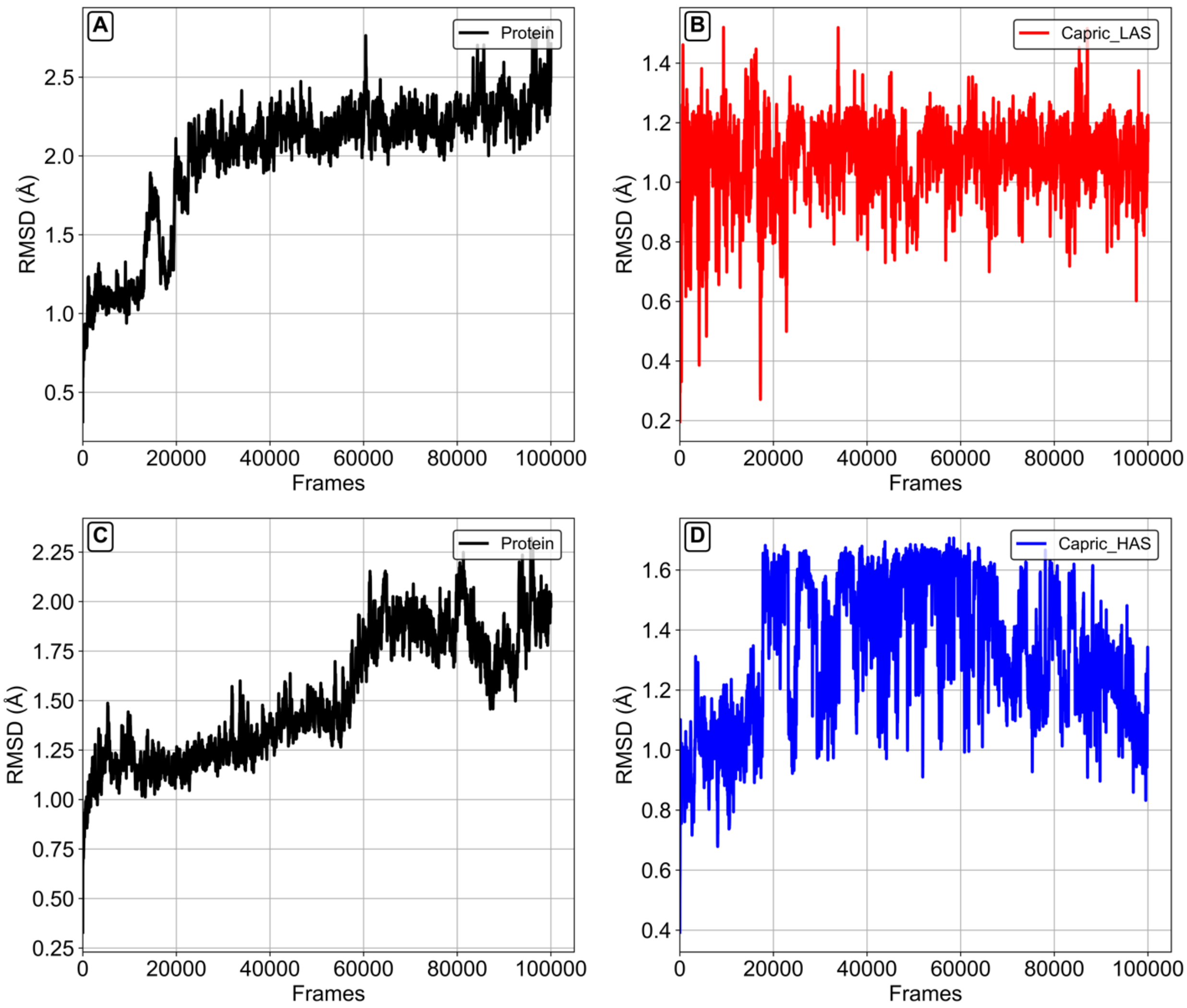

Figure S8: RMSD of Protein (A, C) and Capric acid (B, D) RMSD at LAS and HAS.

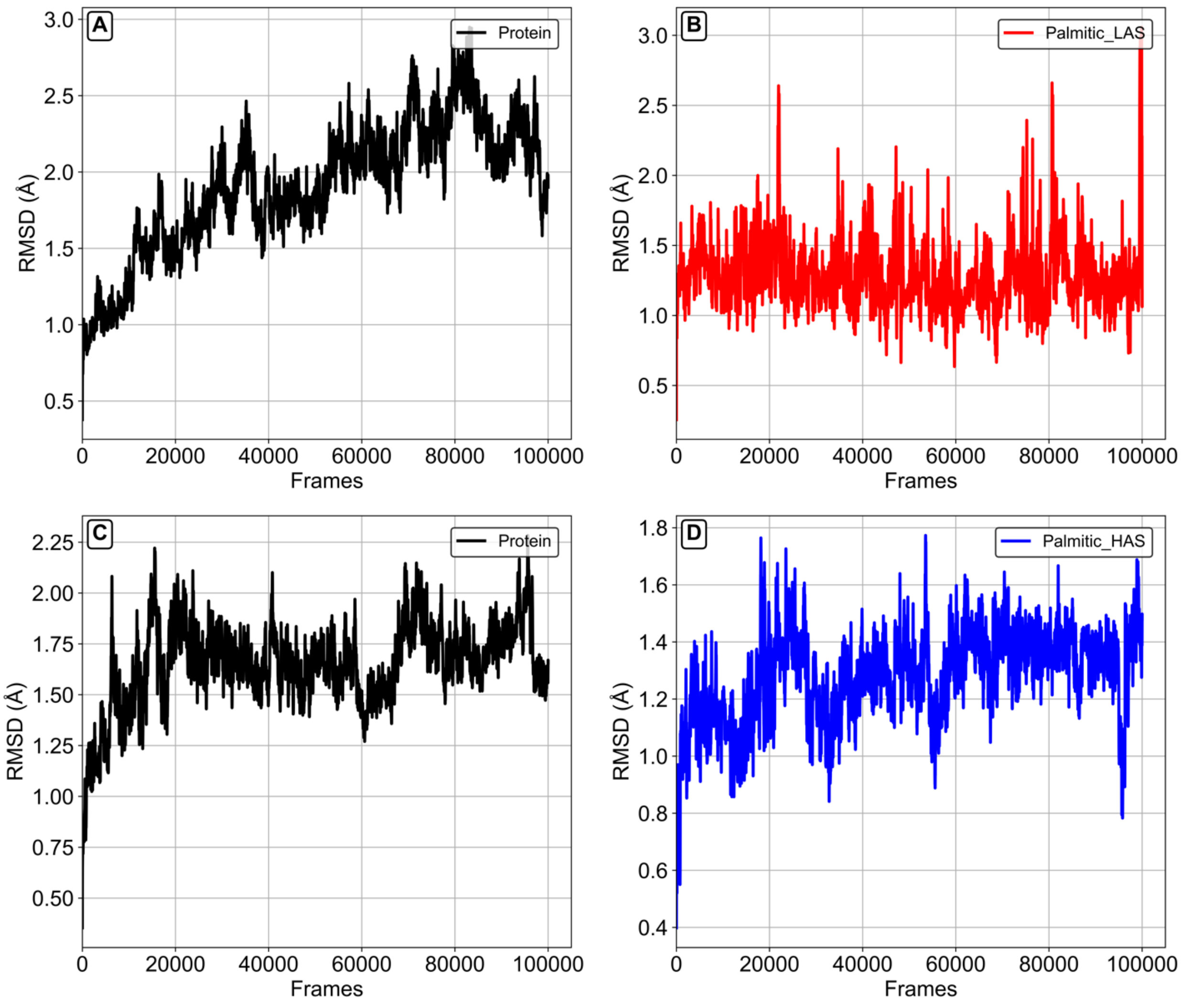

Figure S9: RMSD of Protein (A, C) and Palmitic acid (B, D) RMSD at LAS and HAS.

RMSF

With PFOS isomers at LAS, the root mean square fluctuation (RMSF) values for most of the protein residues ranged from 0.5-2 Å, indicating relatively low fluctuations across most regions of the protein. However, the residues in the L3 loop region (residue numbers:150-200) exhibited higher fluctuations ranging from 3.0-4.5 Å. This increase in fluctuation is primarily attributed to the translocation of PFOS from LAS to HAS. The fluctuation behavior of protein residues is consistent with both linear isomers of PFOS and natural ligands, regardless of the temperature examined. In addition, the residues between 240-250, which form a small loop region connected to the L3 loop, also showed higher fluctuation in a similar range for most cases (**Figure S10**). The RMSF profile of protein residues with ligands at HAS was largely similar to LAS. However, the fluctuations in the L3 loop were reduced by an average of 0.5 Å, likely due to the strong interaction of ligands (PFOS and natural ligands) with the polar residues at HAS. Importantly, for all PFOS isomers, the RMSF of the L3 loop demonstrated higher fluctuations compared to the APO system, highlighting the intrinsic role of the L3 loop for substrate transport. Aside from the L3, all other regions of protein displayed a similar profile for all investigated ligands.

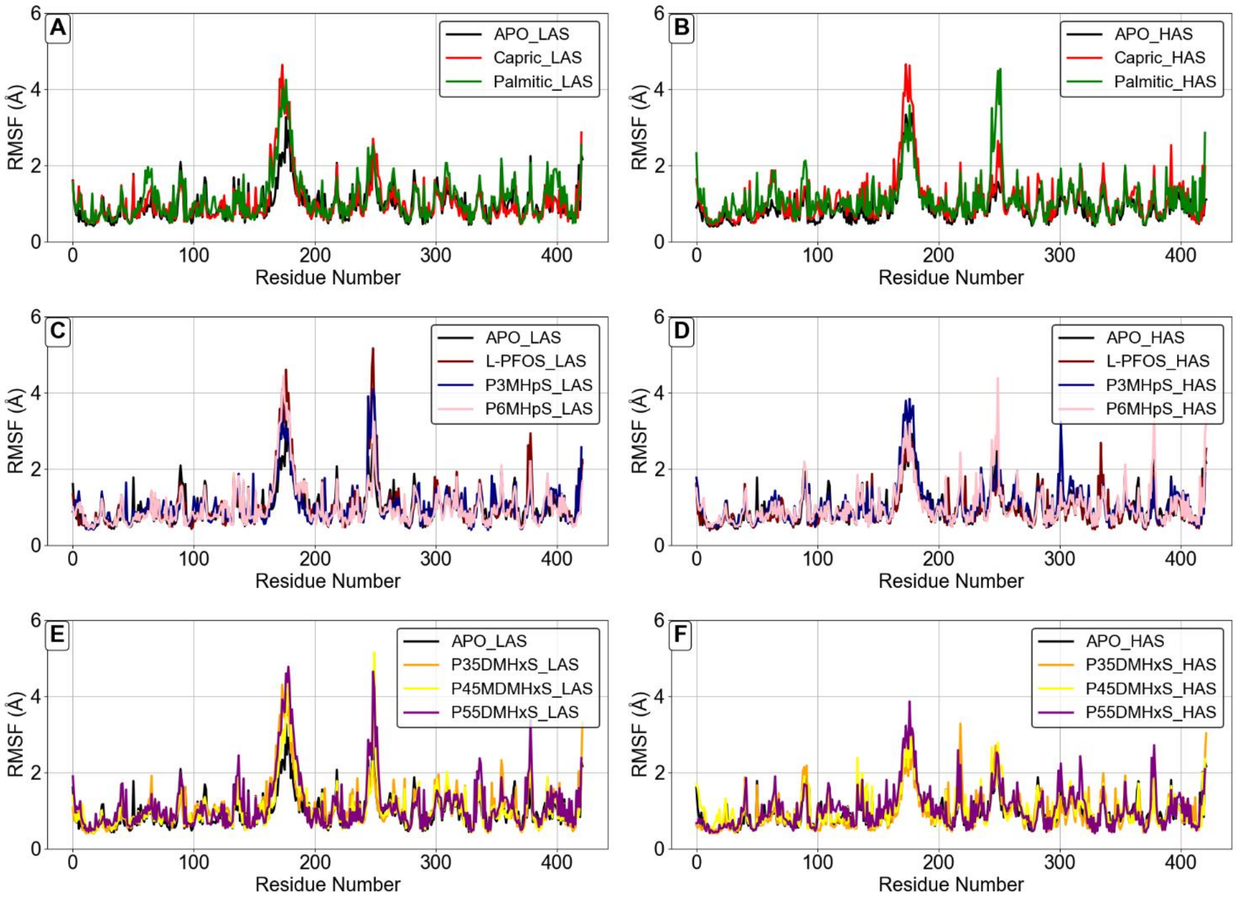

Figure S10: RMSF of FadL where the ligand(s) bind at the LAS (A, C and E) and HAS (B, D and F).

Hydrogen Bonding

a)

b)

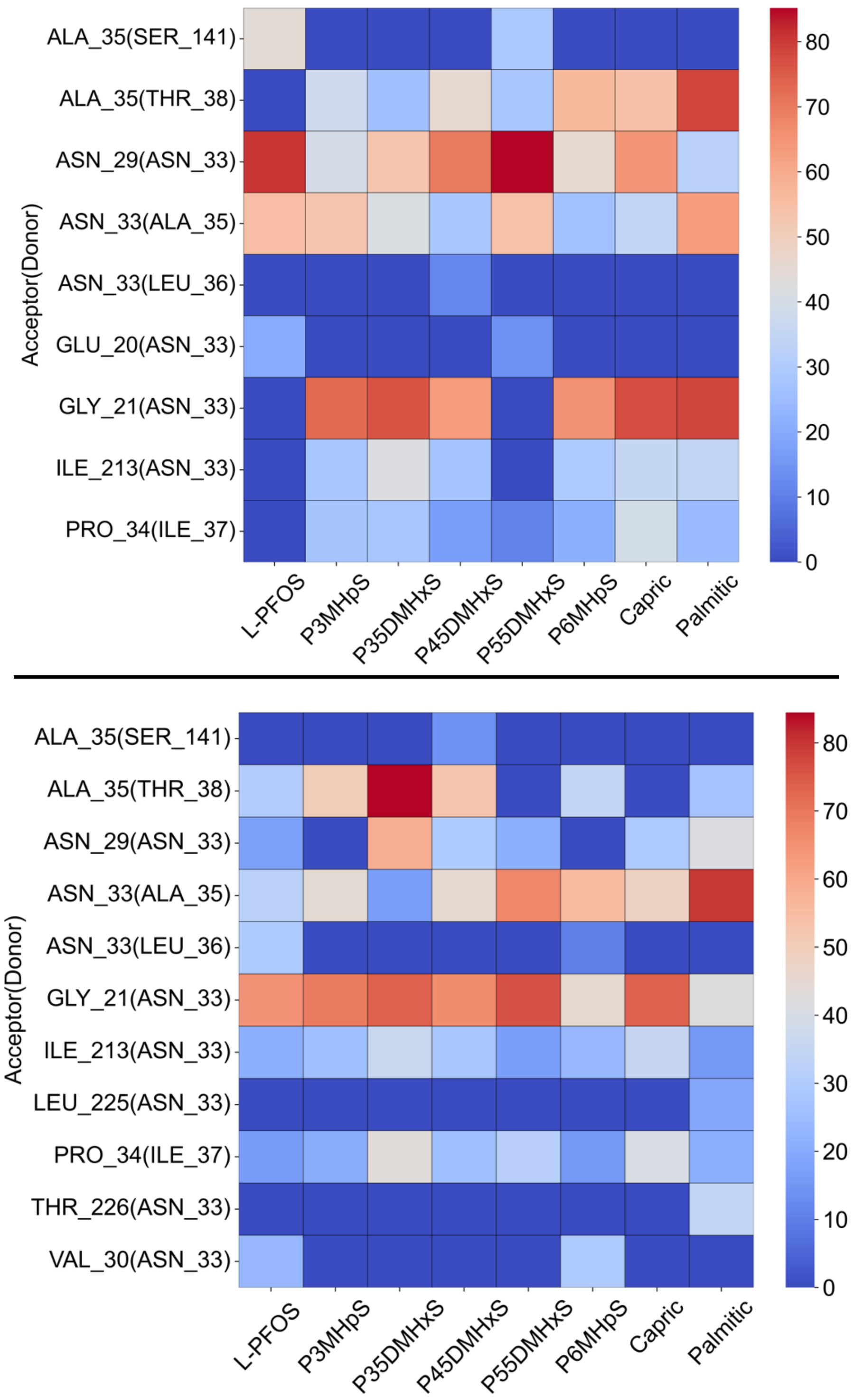

Figure S11: Hydrogen bonding within the NPA sequence of FadL, where the ligand(s) bind at the a) LAS and b) HAS.

VDW Energy

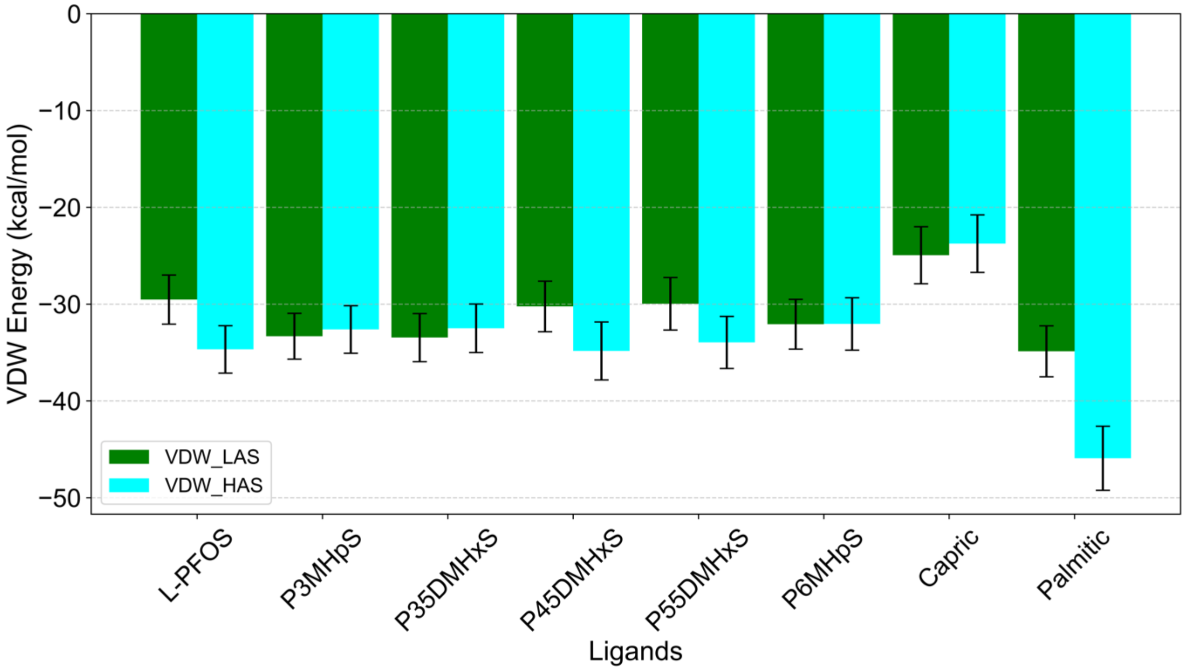

Figure S12: VDW interaction of investigated protein where the ligand(s) bind at the LAS and HAS.

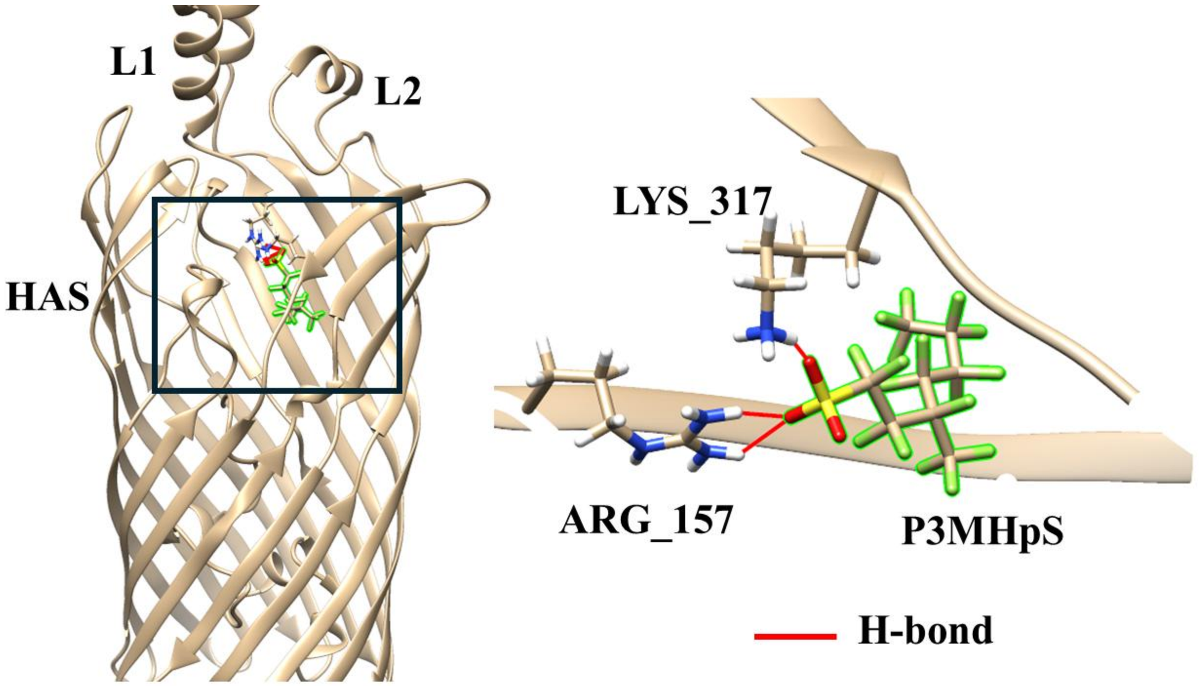

Figure S13: Hydrogen bonding interaction between P3MHpS with neighboring residues at HAS.

Per-residue Decomposition

Residues	L-PFOS	P3MHpS	P35DMHxS	P45DMHxS	P55DMHxS	P6MHpS	Capric	Palmitic
ASP_161	0	0	0	0	0	0	0	0
LEU_162	0	0	0	-1.06	0	-1.09	0	1.16
VAL_166	-1.04	0	0	-1.34	-1.17	-1.41	0	0
THR_187	0	0	0	0	0	0	-1.17	0
ALA_188	0	-1.13	-1.01	0	0	0	0	0
ILE_191	0	-1.18	0	0	0	0	0	0
LEU_243	-1.03	-1.57	-1.51	-1.03	-1.13	0	0	-1.07
ASN_244	0	-2	-1.83	0	0	0	0	0
ASP_161	0	-1.59	-1.27	0	0	0	0	0
ARG_245	0	-1.15	0	0	0	0	0	0
PHE_247	0	0	0	0	0	0	0	0
LEU_252	0	-1.1	0	0	0	0	0	0
PRO_253	0	0	0	-1.31	0	0	0	0

Table S1: Per residue decomposition values (all the values are in **kcal mol^{-1}**) of investigated ligands at LAS

Residues	L-PFOS	P3MHpS	P35DMHxS	P45DMHxS	P55DMHxS	P6MHpS	Capric	Palmitic
ALA_1	-1.34	0	0	0	0	0	0	0
GLU_7	0	0	0	0	0	0	0	0
LEU_104	0	0	0	0	0	0	0	0
VAL_117	0	0	0	1.34	0	0	0	0
THR_121	0	0	-1.59	0	-1.04	0	0	0
ASP_122	0	1.63	1.63	0	1.27	1.28	0	1.67
LEU_123	-1.06	0	0	0	0	0	0	0
ALA_153	0	0	0	0	0	0	0	0
ILE_155	-1.21	-1.69	-1.86	-1.02	-1.35	-1.06	-1.36	-1.79
GLU_156	0	0	0	0	0	0	1.01	0
ARG_157	-2.31	-4.96	-6.76	-4.99	-2.92	-4.03	-10.72	-7.32
LEU_200	0	0	0	0	0	0	0	-1.13
ILE_254	0	0	0	0	-1.04	0	0	0
LEU_267	0	0	0	0	0	0	0	-1.56
THR_268	1.19	0	0	0	0	0	0	0
LEU_304	-1.86	-1.42	-1.51	-1.41	-1.17	-1.64	-1.14	-1.2
PHE_315	0	0	0	0	0	0	0	0
LYS_317	-2.79	-3.66	-4.38	-3.49	-2.48	-2.69	-5.7	-5.8
GLU_319	6.34	1.01	1.69	2.16	1.27	2.75	5.65	5.71
ASP_348	0	0	0	0	0	0	0	0
ARG_357	-1.34	0	0	0	0	0	0	0
SER_360	0	0	0	1.12	1.07	0	0	-1.33
ILE_361	0	0	-1.87	0	-1.07	0	0	0
ASP_363	1.3	0	0	0	0	0	0	0
ASP_365	1.29	0	0	0	0	0	0	0
GLU_399	1.25	1.18	0	2.18	3.2	1.17	1.49	1.4

Table S2: Per residue decomposition values (all the values are in **kcal mol^{-1})** of investigated ligands at HAS

MD Simulations at 310 K

To verify and replicate whether the temperature condition affects the results obtained at 293 K, the FadL–ligand simulations were performed at 310 K. Two ligands were selected for this study, namely L-PFOS and P3MHpS, both of which bind to LAS and HAS.

Binding Energy Calculation

At LAS, P3MHpS exhibited a higher binding energy than L-PFOS under both temperature conditions. No significant difference was observed between the two temperatures for each ligand. However, L-PFOS showed a decrease of ~3 kcal mol^{-1} in binding energy at 310 K, which can be attributed to a higher number of residues maintaining attractive interactions compared to 293 K (**Figure S14 and Table S3**).

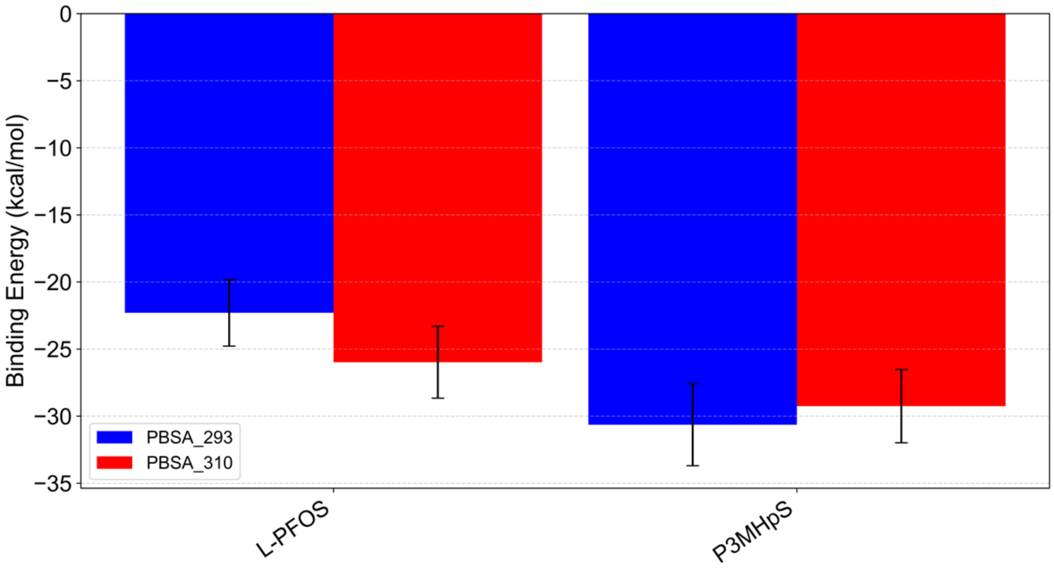

Figure S14: Binding Energy of ligands investigated at LAS

At the HAS site, P3MHpS displayed a higher binding energy than L-PFOS. Notably, significant differences in binding energies were observed under different temperature conditions: at 310 K, the binding energy of linear PFOS increased by -6 kcal mol^{-1}, whereas for P3MHpS, it decreased by 7 kcal mol^{-1}. In the case of PFOS, this change was primarily due to a reduction of approximately 5 kcal mol^{-1}in repulsive interactions with GLU_319 at the elevated temperature. In contrast, the decrease in binding energy for P3MHpS was attributed to reduced attractive interactions with neighboring residues and alterations in its hydrogen bonding pattern (**Figure S15 and Table S4**).

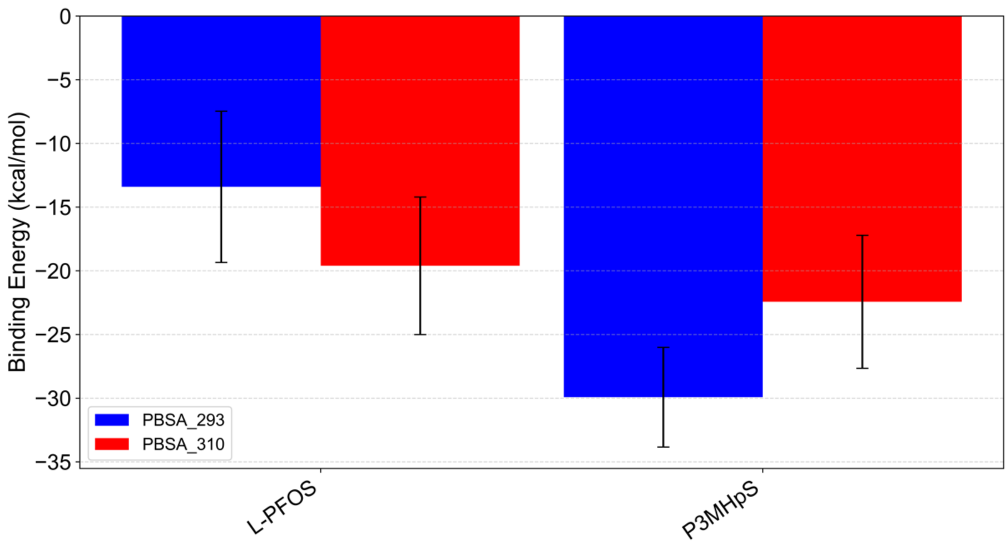

Figure S15: Binding Energy of ligands investigated at HAS

Hydrogen Bonding

No significant hydrogen bonding interactions were observed for the ligands at LAS, as this site contains fewer polar residues. As a result, L-PFOS did not show any considerable hydrogen bonding at LAS for either temperature (**Figure S16)**. In contrast, P3MHpS maintained hydrogen bonding with the amide group of ASN_244 at both temperatures; however, the hydrogen bonding percentage decreased by ~25% at 310 K. This reduction is associated with a decreased attractive contribution from ASN_244, which dropped from ~ -2 kcal mol^{-1}at 293 K to ~ -1 kcal mol^{-1} at 310 K (**Table S3**), which likely explains the observed reduction in hydrogen bonding. Additionally, a significant change was observed with ARG_245, where hydrogen bonding dropped from 40% to less than 5%. This sudden change can be explained by examining the per-residue decomposition data, where the attractive contribution of P3MHpS towards ARG_245 is approximately -1 kcal mol^{-1} at 293 K, which is missing at higher temperatures (**Table S3**).

A significant difference in the hydrogen bonding pattern is observed for both ligands at higher temperatures, particularly for P3MHpS. As shown in **Figure S16**, P3MHpS exhibits a 40% reduction in hydrogen bonding with ARG_157 at higher temperatures. This change can be attributed to a nearly 50% decrease in attractive interactions with ARG_157 (**Table S4**). On the other hand, P3MHpS establishes an interaction with SER_358 by ~ 26%, which is not observed at lower temperatures. In the case of L-PFOS, the hydrogen bonding with SER_360 is almost non-existent at higher temperatures, while there is a slight increase of 10% in hydrogen bonding with ARG_157 at 310 K. This change in hydrogen bonding pattern at HAS is likely due to the increased fluctuation of residues at higher temperatures.

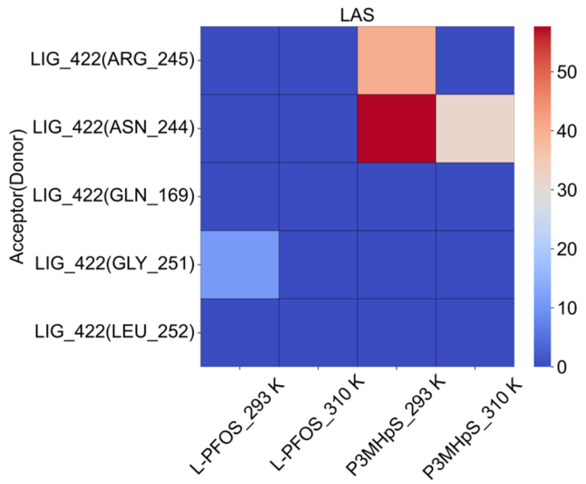

Figure S16: Hydrogen bonding of investigated ligands at LAS and HAS

On the other hand, the hydrogen bonding within the NPA sequence (**Figure S17**) showed no significant differences between the two temperatures, particularly between GLY_21 and ASN_33.

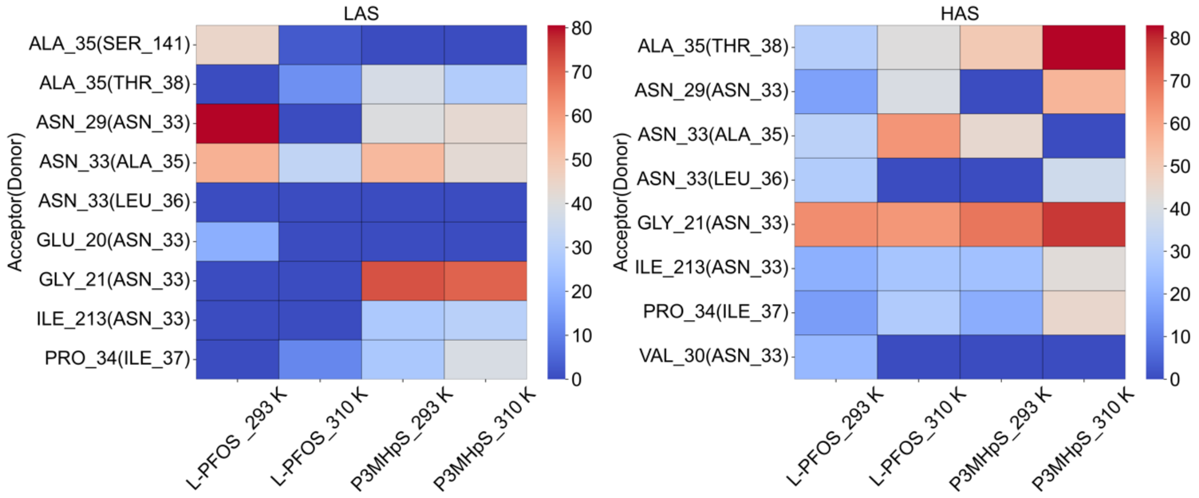

Figure S17: Hydrogen bonding within the NPA sequence of FadL, where the ligand(s) bind at the LAS and HAS

Per-residue decomposition

From the per-residue decomposition data (**Table S3**), considerable differences were observed between the two temperatures. At LAS. L-PFOS demonstrated a more attractive contribution at the higher temperature, which may be one of the contributing factors for the increase in binding energy. In contrast, P3MHpS followed a similar overall trend, with only a few exceptions such as interaction between ASP_161 and ARG_245 are completely missing at higher temperature. In

contrast, at higher temperature P3MHpS showed an attractive interaction with THR_187, which is missing at lower temperature. For HAS (**Table S4**), notable differences between the two temperatures. At higher temperatures, the repulsive contribution for L-PFOS was reduced. Conversely, for P3MHpS, the overall attractive interaction decreased to 310 K, particularly with ARG_157 and LYS_317.

**Residues**	**L-PFOS_293 K**	**L-PFOS_310 K**	**P3MHpS_293 K**	**P3MHpS_310 K**
VAL_166	-1.04	0	0	0
THR_187	0	0	0	-1.21
ALA_188	0	-1.1	-1.13	-1.16
ILE_191	0	-1.53	-1.18	-1.57
LEU_243	-1.03	-1.03	-1.57	-1.8
ASN_244	0	0	-2	-1.02
ASP_161	0	0	-1.59	0
ARG_245	0	0	-1.15	0
PHE_247	0	-1.08	0	0
LEU_252	0	0	-1.1	-1.11

Table S3: Per residue decomposition values (all the values are in **kcal/mol)** of investigated ligands at LAS

**Residues**	**L-PFOS_293 K**	**L-PFOS_310 K**	**P3MHpS_293 K**	**P3MHpS_310 K**
ALA_1	-1.34	0	0	0
ASP_122	0	1.2	1.63	1.35
LEU_123	-1.06	0	0	-1.13
ILE_155	-1.21	-1.56	-1.69	0
ARG_157	-2.31	-1.01	-4.96	-2.29
LEU_304	-1.86	0	-1.42	-1.15
LYS_317	-2.79	-2.27	-3.66	-2.78
GLU_319	6.34	1.51	1.01	0
ARG_357	-1.34	0	0	-1.07
ASP_365	1.29	0	0	0
GLU_399	1.25	1.59	1.18	0

Table S4: Per residue decomposition values (all the values are in **kcal/mol)** of investigated ligands at HAS
